# Aryl Azides as Phosphine-Activated Switches for Small Molecule Function

**DOI:** 10.1038/s41598-018-37023-6

**Published:** 2019-02-06

**Authors:** Bradley Lukasak, Kunihiko Morihiro, Alexander Deiters

**Affiliations:** Department of Chemistry, University of Pittsburgh, 219 Parkman Ave, Pittsburgh, PA 15260 USA

## Abstract

Engineered small molecule triggers are important tools for the control and investigation of biological processes, in particular protein function. Staudinger reductions of aryl azides to amines through the use of phosphines can trigger an elimination reaction, and thereby activation of a functional molecule, if an appropriately positioned leaving group is present. We conducted detailed investigations of the effect of aryl azide and phosphine structure on both the mechanism and kinetics of these reaction-induced eliminations and identified phosphine/azide pairs that enable complete activation within minutes under physiologically relevant conditions.

## Introduction

Temporal control over the activation of small (and large) molecules in biological settings is a valuable tool in the study of physiological processes. The use of small molecule triggers provides a promising approach in which a protecting group is installed onto a biologically active molecule, rendering it inactive, and enabling deprotection and activation by a small molecule trigger^1,2^. The applications of small molecule-triggered deprotection strategies include cell imaging^3,4^, gene activation^5^, and control of protein function^6–11^. Furthermore, recent interest has sparked the development of chemically activatable prodrugs of anticancer agents^12–17^, antibody-drug conjugates^18^, and nucleic acid-based therapeutics^19,20^. These approaches harness bioorthogonal reactions such as azide reduction^4,6,9^, boron oxidation^3,5,20^, metal-catalyzed deprotection^8,10,13,15,16^, and tetrazine ligation^7,11,12,14,17–19^. Some of them have been demonstrated to be compatible with biological systems – not only in living cells, but also in animals^11–13,16–18^. To further expand the utility of small molecule-triggered reactions, optimization of the reaction pair with rapid kinetics and high release yields is required^21^.

**Figure 1:**
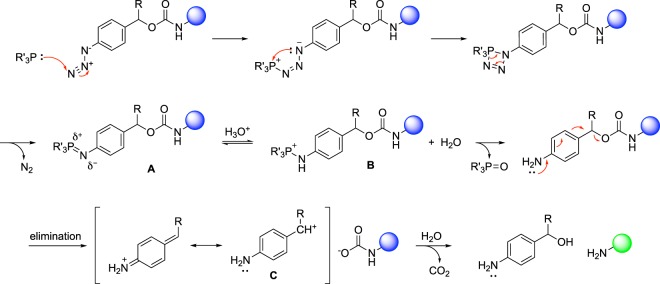
Coupling of the Staudinger reduction and 1,6-elimination for the activation (green sphere) of previously inactive molecules (blue sphere). The aromatic azide is stable until reduction through phosphine treatment, followed by 1,6-elimination and carbamic acid decarboxylation. R = H, Me, or Ph.

Here, we are reporting a systematic study of a chemical trigger based on the Staudinger reduction (Fig. 1), using a set of aryl azides treated with a set of aliphatic and aromatic phosphines. The reduction of an aryl azide to an aniline results in spontaneous 1,6- or 1,4-eliminations in the presences of a suitable leaving group^22^. The resulting iminoquinone methide cation intermediate is rapidly quenched by water. If the leaving group is a carbamate, spontaneous decarboxylation yields a free amine in the released molecule.

In addition to utilizing a Staudinger reduction in the formation of the aromatic amine, other reductions, which are less likely to be compatible with a cellular environment, have been used in this context, such as nitro reductions utilizing zinc^23^, and azide reductions utilizing dithiothreitol^24^ and glutathione (very slow reactivity at low concentrations)^25^. In addition to the well-established orthogonality of aryl phosphines (which do not reduce disulfide bonds)^26^ and azides to other cellular chemistries^27,28^, the Staudinger reduction-based trigger affords high specificity and efficiency of activation at low concentrations; however, it has only infrequently been applied^9,29^. We suspect that this is, in part, due to a lack of design rules for an optimized azide/phosphine pair and the corresponding mechanistic and kinetic details of the fragmentation reaction.

## Results

**Figure 2:**
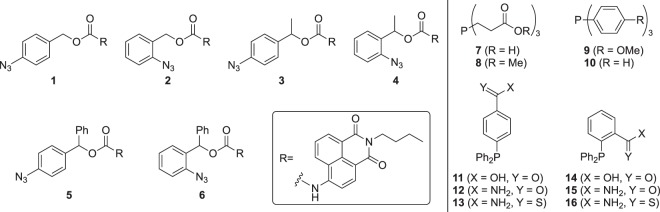
Aryl azides and phosphines used in this Staudinger reduction triggered elimination study, leading to the release of a ABNI small molecule as a proof-of-concept.

Since the kinetics of the overall activation cascade are dependent upon the structure of the azide and the phosphine, we synthesized a panel of azide-containing protecting groups (**1**–**6**) and a panel of phosphine small molecule triggers (**7**–**16**) (Fig. 2; see Supporting Information for synthetic schemes and detailed synthetic protocols). Different aryl-azides were selected in order to study the effect of an α-substituent, stabilizing a transiently formed iminoquinone methide cation^30^, and to investigate the role of a *para-* versus an *ortho*-position of the azido group. Using different phosphines, the effect of differing electron density at the phosphorous center on the nucleophilic attack onto the azide or on aza-ylide hydrolysis was studied. Additionally, coordinating functional groups on the phosphine may accelerate the reaction by promoting hydrolysis and/or elimination^31^. For a fluorescence-based readout, we applied a 4-amino-*N*-butyl-1,8-naphthalimide (ABNI) fluorophore which was deactivated through protection via a carbamate linkage^32,33^. Upon treatment with a phosphine and subsequent cleavage of the protecting group, the fluorophore is activated allowing the reaction to be readily monitored.

**Figure 3:**
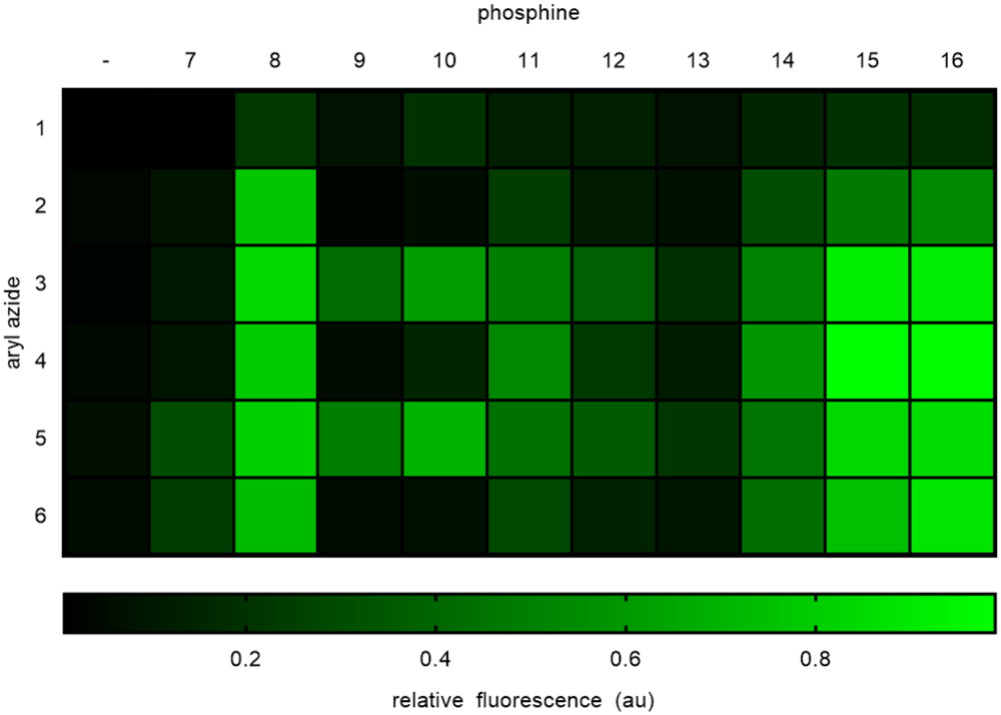
Initial screening of phosphines (100 μM) and aryl azides (10 µM). Fluorescence was measured 1 h after phosphine treatment. Conversion to active ABNI was determined by comparing to fluorescence of independently synthesized ABNI (10 μM). The heat map shows percent conversion of azide to active ABNI with black representing 0% to 20% conversion and bright green representing 80% to 100% conversion.

To rapidly screen all azide/phosphine combinations, fluorophore activation was conducted in PBS/DMSO (4:1; pH 7.4) in a multi-well format and fluorescence was measured at a single time-point (60 min) (Fig. 3). The assay was conducted in triplicate and deprotection conversions were determined based on fluorescence normalized to the non-protected ABNI. In the absence of α-substituents, reactivity was diminished which we hypothesize is due to the elimination being rate limiting in these cases. In general, α-substituents such as phenyl or methyl groups on the aryl azides resulted in increased product formation, but displayed significant dependence on phosphine structure because now the azide reduction became the rate-limiting step (as further investigated below).

The azides **1** and **2** both lack an α-substituent and their reactivity appeared to be less dependent on phosphine identity compared to other azides (Supporting Fig. S1). Because the only variable is the presence or absence of an α-substituent, the reactivity difference can be explained by the rate limiting step being either the 1,6- or the 1,4-elimination for azides **1** and **2**. The presence of an electron donating α-substituent in **3**–**6** makes the formation of the cation intermediate **C** more favorable (Fig. 1)^34^. This increases the rate of the reaction making the reduction to the aniline the rate-limiting step for azides **3**–**6**. The identity of the α-substituent did not play a major role in the relative amount of product formation. While phenyl groups are known to be better stabilizers of carbocations^35^, in this reaction the stabilization of the carbocation intermediate by the methyl group is enough to switch the rate-limiting step. Therefore, we saw comparable amounts of product formation for azides **3**–**6**. Thus, not surprisingly, phosphine identity impacted the rate-limiting step and thus the rate of product formation for azides **3**–**6** deprotection.

The two trialkyl phosphines **7** and **8** displayed different reactivity, compared to the aromatic phosphines. Deprotection with **7** resulted in very low levels of elimination, whereas **8** provided fast fluorophore activation followed by a sharp decrease in reaction rate. We posit that this is a result of trialkyl phosphine susceptibility to oxidation under mimicked physiological conditions (aqueous environment, pH 7.4, open to ambient air). In contrast, analysis of the reaction mixture for reduction using the aromatic phosphines **9** by LC/MS-analysis showed a strong signal for the aza-ylide intermediate (Supporting Figs S2 and S3). Thus, the rate-limiting step for the reaction was the aza-ylide hydrolysis when using electron-rich triaryl phosphines. Both triaryl phosphines **9** and **10** showed very similar reactivity patterns such that when paired with *ortho*-azides (**4** and **6**), only a minimal amount of elimination occurred; possibly due to the steric bulk of an aza-ylide in the *ortho*-position hindering hydrolysis. Pairing with the *para*-azides **3** and **5**, however, resulted in good elimination rates, with **10** performing better than **9**. Both **9** and **10** can stabilize the positive charge that develops on the phosphorous in the protonated intermediate **B** (Fig. 1). This stabilization lowers the electrophilicity of the phosphorous and may make the aza-ylide less prone to hydrolysis. This led us to hypothesize that the reaction should have a dependence on the pH of the reaction. Not surprisingly, rate enhancement was achieved when the reaction of the azide **3** with the phosphine **9** was performed under basic conditions (Supporting Fig. S12). The presence of a better nucleophile (hydroxide) in higher concentration compensated for the lower electrophilicity of the phosphorous caused by positive charge stabilization by the (electron-rich) aromatic rings. Importantly, this rate enhancement was only achieved for *para*-azides indicating that the *ortho*-aza-ylide intermediate is too sterically hindered to undergo faster hydrolysis even in the presence of basic conditions (Supporting Fig. S13).

In contrast, the remaining phosphines contained groups that were electron withdrawing but varied in their position on the aromatic rings. Like **9** and **10**, the phosphines **11** and **12** also formed stable aza-ylides, as confirmed by LC/MS (Supporting Figs S4–S7). Fluorophore activations that are initiated with azide reductions using **11**, **12**, and **13** display an altered equilibrium between intermediates **A** and **B** (Fig. 1). We hypothesize that the protonation to yield the intermediate **B** is necessary for, and precedes, aza-ylide hydrolysis. ^31^P-NMR revealed the following order of electron-withdrawing ability of substituents in the 4-position: carboxyl < carboxamide < thiocarboxamide (see Supporting Table S1). We speculate that less electron withdrawing substituents (**11**) push the equilibrium to the right and stronger electron withdrawing groups (**13**) push the equilibrium to the left. In all three cases, the hydrolysis is fast because the positively charged and more electrophilic phosphorous facilitates attack by water. The enhanced electrophilicity in the protonated intermediate overcomes any steric hindrance that could otherwise obstruct hydrolysis of *ortho*-azides. These observations are further supported by a rate enhancement when the reactions were carried out under acidic conditions, further facilitating protonation (Supporting Figs S14 and S15). We used phosphines **11** and **12** to show that the stronger the electron withdrawing group, the more of an effect that acidic conditions have on the reaction.

The *ortho*-substituted phosphines **14**, **15**, and **16** displayed an upfield ^31^P-NMR shift and a higher concentration of the elimination product at the 60 minute time point. The ^31^P-NMR spectrum was not impacted by the strength of the electron-withdrawing group, but rather the potential for functional group coordination to the phosphorous, since the signals for **15** and **16** were shifted upfield with respect to their *para* counterparts indicating more electron density at the phosphorous center. Phosphine **14** showed a lower degree of coordination to the phosphorous.

**Figure 4:**
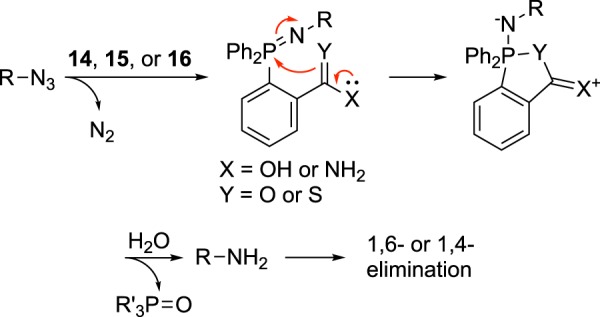
Proposed reaction mechanism for coordinating phosphines.

Phosphines that undergo neighboring group participation (Fig. 4) resulted in a predictable set of LC/MS results where both azides **3** and **4** reacted in an identical fashion (Supporting Figs S8–S11) where only starting material and product and no reaction intermediates (e.g., aza-ylides) were observed (in contrast to other azide/phosphine pairs discussed earlier). Due to rapid aza-ylide hydrolysis through neighboring-group participation, the rate-limiting step of the Staudinger reduction is presumably the attack of the phosphine onto the azide. The lack of intermediates at high concentration is ideal for physiological studies as it will minimize side reactions with possible biological substrates.

**Figure 5:**
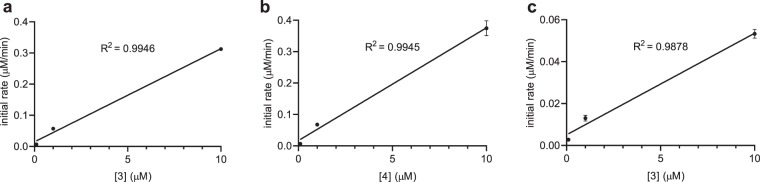
Pseudo-first order kinetics from plotting initial rate vs. azide concentration utilizing three initial azide concentrations (0.1 μM, 1 μM, and 10 μM). The phosphine was used in excess and its concentration assumed constant. (**a**) Azide **3** deprotected with phosphine **15**. (**b**) Azide **4** deprotected with phosphine **15**. (**c**) Azide **3** deprotected with phosphine **9**. All reactions displaying a linear trend for its rate with respect to concentration of azide, and all reactions were first order with respect to azide concentration. Error bars represent standard deviations generated from three independent experiments.

To identify the reaction order on the azide under physiological concentrations, initial rate studies were performed using azides **3** and **4** paired with the phosphine **15** and the azide **3** paired with the phosphine **9**. As in previous experiments, we wanted to show that for electron poor phosphines such as **15**, the ring position of the azido group does not impact the rate law. We only used azide **3** for phosphine **9** due to poor reaction rates for *ortho*-azides when paired with electron rich phosphines. We used the phosphines **9** and **15** in these studies because they display different rate limiting steps but posited that the azide concentration would impact the rate of the reaction for both. In biological experiments, the phosphine triggers will be present in comparatively high concentrations (up to low mM concentrations depending on solubility and cellular toxicity)^9^ and thus reaction kinetics can be approximated by pseudo-first order kinetics. Initial rates were calculated using fluorescence data (see Supporting Table S2) and plotted versus azide concentration (Fig. 5). By using three different azide concentrations, we discovered a linear relationship between the initial reaction rate and the initial azide concentration indicating a first order dependence. Thus, the faster the reduction to the aniline the faster the overall reaction will proceed. This leads to the conclusion that phosphines that undergo neighboring group participation, such as phosphine **15**, are optimal for triggering 1,6 or 1,4-eliminations for azido benzyl carbamates.

## Discussion

We have performed a detailed study to identify factors that influence the rate of a Staudinger reduction initiated 1,4 or 1,6-elimination. In general, α-substituents resulted in increased reaction rates presumably due to formation of a stable cation intermediate. There was no significant difference between methyl-substitution and phenyl-substitution based on fluorophore activation experiments. The position of the azide (*para* vs *ortho*) on the aromatic ring played a role depending on the identity of the phosphine utilized. Electron rich phosphines such as **9** favored *para* azides and electron poor phosphines such as **11** did not display a significant preference to the azide position. We determined this was due to a change in the rate limiting step, whereby electron-rich phosphines are limited by the hydrolysis of the aza-ylide which is sterically hindered when this intermediate is in the *ortho*-position with respect to the starting azido group. For electron-poor phosphines, the phosphorous center was more electrophilic and thus was limited by the protonation of the aza-ylide intermediate, a step that is not impacted by sterics. Unsurprisingly, phosphine identify ultimately had the largest impact on efficient activation, and phosphines with *ortho*-groups capable of coordinating to the phosphorous center had a clear kinetic advantage. Both *para*- and *ortho*-azides with α-substituents, with the methyl group displaying slightly better reactivity, reacted rapidly with the phosphines **15** and **16** displaying pairs that are optimal for studies where the release of a functional molecule is desired to be fast and efficient. Importantly, such pairs also operate at low concentrations (<1 μM) of the azide trigger.

These results have the potential to be utilized in future studies where fast and efficient release of a molecule of interest is desired. Placing control of biologically relevant molecules under the release of an exogenous small molecule allows for precise temporal control over the process of interest. Our system is ideal for such investigations due to its bioorthogonality. In summary, this study has allowed the optimization of phosphine-triggered eliminations for the activation of small molecules, in particular a fluorophore; however, the results are most likely extendable to the activation of biological macromolecules, e.g., proteins, through Staudinger reduction.

## Methods

### General Staudinger reduction assays

Staudinger reduction assays were performed by incubating azide (10 μM) with phosphine (100 μM) in a 4:1 mixture of PBS (137 mM NaCl, 2.7 mM KCl, 10 mM Na_2_HPO_4_, 1.8 mM KH_2_PO_4_, pH 7.4) and DMSO. Azide’s, phosphines, and free ABNI were stored as 10x stocks in DMSO with respect to final assay concentrations. Assays were carried out in 384-well format in a total reaction volume of 50 μL and allowed to proceed for 1 hour at room temperature. After 1 hour, fluorescence was measured using a plate reader (λ_ex_ = 420 nm, λ_em_ = 540 nm). All fluorescence values were set relative to a positive control which consisted of 10 μM ABNI in assay buffer.

### Concentration dependent Staudinger reduction

To assess the effect of azide concentration on reaction rates, the general Staudinger reduction assay (see above) was utilized with slight alterations. In brief, the final concentration of azide was varied to be 0.1 μM, 1 μM, and 10 μM while all other assay conditions were kept constant. Fluorescence was measured every 2 minutes for 1 hour using a plate reader (λ_ex_ = 420 nm, λ_em_ = 540 nm). All fluorescence values were set relative to a positive control which consisted of 10 μM ABNI in assay buffer. The initial rate of the reactions was calculated and plotted against the initial concentration of azide.

### General LC/MS analysis protocol

To assess the formation of reaction intermediates, the general Staudinger reduction assay (see above) was utilized with slight alterations. In brief, the total reaction volume was increased to 200 μL and the reaction was analyzed by LC/MS after 30 minutes utilizing a gradient of 10–90% acetonitrile with 0.1% Formic Acid.

### General pH-dependence assay protocol

To assess the effect of pH on the rate of reaction, the general Staudinger reduction assay (see above) was utilized with slight alterations. In brief, in place of PBS, Citrate Buffer (pH 5.0) and glycine-NaOH (pH 9.0) were utilized to allow for study of the reactions under acidic and basic conditions. Fluorescence was measured every 10 minutes for 1 hour using a plate reader (λ_ex_ = 420 nm, λ_em_ = 540 nm). All fluorescence values were set relative to a positive control which consisted of 10 μM ABNI in assay buffer.

## Supplementary information

Aryl Azides as Phosphine-Activated Switches for Small Molecule Function

## Data Availability

All data generated or analyzed during this study are included in this published article (and its Supplementary Information files).

## References

[1] Shieh P, Bertozzi CR (2014). Design strategies for bioorthogonal smart probes. Org. Biomol. Chem..

[2] Li J, Chen PR (2016). Development and application of bond cleavage reactions in bioorthogonal chemistry. Nat. Chem. Biol..

[3] Kim J, Bertozzi CR (2015). A bioorthogonal reaction of N-oxide and boron reagents. Angew. Chem. Int. Ed..

[4] Xue ZW (2018). Organelle-directed staudinger reaction enabling fluorescence-on resolution of mitochondrial electropotentials via a self-immolative charge reversal probe. Anal. Chem..

[5] Govan JM, McIver AL, Riggsbee C, Deiters A (2012). Hydrogen peroxide induced activation of gene expression in mammalian cells using boronate estrone derivatives. Angew. Chem. Int. Ed..

[6] Ge Y, Fan XY, Chen PR (2016). A genetically encoded multifunctional unnatural amino acid for versatile protein manipulations in living cells. Chem. Sci..

[7] Li J, Jia S, Chen PR (2014). Diels-Alder reaction-triggered bioorthogonal protein decaging in living cells. Nat. Chem. Biol..

[8] Li J (2014). Palladium-triggered deprotection chemistry for protein activation in living cells. Nat. Chem..

[9] Luo J, Liu QY, Morihiro K, Deiters A (2016). Small-molecule control of protein function through Staudinger reduction. Nat. Chem..

[10] Wang J (2016). Palladium-triggered chemical rescue of intracellular proteins via genetically encoded allene-caged tyrosine. J. Am. Chem. Soc..

[11] Zhang G (2016). Bioorthogonal chemical activation of kinases in living systems. ACS Central Sci..

[12] Oneto JMM, Khan I, Seebald L, Royzen M (2016). *In vivo* bioorthogonal chemistry enables local hydrogel and systemic pro-drug to treat soft tissue sarcoma. ACS Central Sci..

[13] Perez-Lopez AM (2017). Gold-triggered uncaging chemistry in living systems. Angew. Chem. Int. Ed..

[14] Versteegen RM, Rossin R, ten Hoeve W, Janssen HM, Robillard MS (2013). Click to release: instantaneous doxorubicin elimination upon tetrazine ligation. Angew. Chem. Int. Ed..

[15] Volker T, Dempwolff F, Graumann PL, Meggers E (2014). Progress towards bioorthogonal catalysis with organometallic compounds. Angew. Chem. Int. Ed..

[16] Weiss JT (2014). Extracellular palladium-catalysed dealkylation of 5-fluoro-1-propargyl-uracil as a bioorthogonally activated prodrug approach. Nat. Commun..

[17] Tu JL, Xu MH, Parvez S, Peterson RT, Franzini RM (2018). Bioorthogonal removal of 3-isocyanopropyl groups enables the controlled release of fluorophores and drugs *in vivo*. J. Am. Chem. Soc..

[18] Rossin R (2016). Triggered drug release from an antibody-drug conjugate using fast “click-to-release” chemistry in mice. Bioconjugate Chem..

[19] Khan I, Seebald LM, Robertson NM, Yigit MV, Royzen M (2017). Controlled in-cell activation of RNA therapeutics using bond-cleaving bio-orthogonal chemistry. Chem. Sci..

[20] Mori S, Morihiro K, Okuda T, Kasaharaa Y, Obika S (2018). Hydrogen peroxide-triggered gene silencing in mammalian cells through boronated antisense oligonucleotides. Chem. Sci..

[21] Fan XY (2016). Optimized tetrazine derivatives for rapid bioorthogonal decaging in living cells. Angew. Chem. Int. Ed..

[22] Alouane A, Labruere R, Le Saux T, Schmidt F, Jullien L (2015). Self-immolative spacers: kinetic aspects, structure-property relationships, and applications. Angew. Chem. Int. Ed..

[23] Warnecke A, Kratz F (2008). 2,4-bis(hydroxymethyl)aniline as a building block for oligomers with self-eliminating and multiple release properties. J. Org. Chem..

[24] Griffin RJ (1996). The 4-azidoberazyloxycarbonyl function; Application as a novel protecting group and potential prodrug modification for amines. J. Chem. Soc., Perkin Trans..

[25] Kinski E (2016). 4-Azidobenzyl ferrocenylcarbamate as an anticancer prodrug activated under reductive conditions. J. Inorg. Biochem..

[26] Schlieve CR (2006). Synthesis and characterization of a novel class of reducing agents that are highly neuroprotective for retinal ganglion cells. Exp. Eye. Res..

[27] Agard NJ, Baskin JM, Prescher JA, Lo A, Bertozzi CR (2006). A comparative study of bioorthogonal reactions with azides. ACS Chem. Biol..

[28] Sletten EM, Bertozzi CR (2011). From mechanism to mouse: a tale of two bioorthogonal reactions. Acc. Chem. Res..

[29] Pothukanuri S, Winssinger N (2007). A highly efficient azide-based protecting group for amines and alcohols. Org. Lett..

[30] Alajarin M, Marin-Luna M, Ortin MM, Sanchez-Andrada P, Vidal A (2009). Benzylic Newman-Kwart rearrangement of O-azidobenzyl thiocarbamates triggered by phosphines: pseudopericyclic [1,3] shifts via uncoupled concerted mechanisms. Tetrahedron.

[31] Saneyoshi H (2014). Triphenylphosphinecarboxamide: an effective reagent for the reduction of azides and its application to nucleic acid detection. Org. Lett..

[32] Wu J (2013). Thiol-inducible direct fluorescence monitoring of drug release. Org. Biomol. Chem..

[33] Morihiro K, Ankenbruck N, Lukasak B, Deiters A (2017). Small molecule release and activation through DNA computing. J. Am. Chem. Soc..

[34] Richard JP, Amyes TL, Bei L, Stubblefield V (1990). The effect of beta-fluorine substituents on the rate and equilibrium-constants for the reactions of alpha-substituted 4-methoxybenzyl carbocations and on the reactivity of a simple quinone methide. J. Am. Chem. Soc..

[35] Olah GA, Spear RJ (1975). Stable carbocations. 180. C-13 and H-1 nuclear magnetic-resonance spectroscopic study of phenyl-substituted, methyl-substituted, and cyclopropyl-substituted alkenyl (Allyl) cations - further studies of trend of charge-distribution and relative delocalization afforded by phenyl, methyl, and cyclopropyl groups. J. Am. Chem. Soc..

