# Supplementary material for: Aryl Azides as Phosphine-Activated Switches for Small Molecule Function
Source: Sci Rep. 2019 Feb 6;9:1470. doi: 10.1038/s41598-018-37023-6 (PMC6365568; doi:10.1038/s41598-018-37023-6)
Supplement: Supplementary file 1 — Aryl Azides as Phosphine-Activated Switches for Small Molecule Function [file 41598_2018_37023_MOESM1_ESM.pdf]

SUPPORTING INFORMATION

Aryl Azides as Phosphine-Activated Switches for Small Molecule Function

Bradley Lukasak, Kunihiko Morihiro, and Alexander Deiters*

University of Pittsburgh, Department of Chemistry, 219 Parkman Ave, Pittsburgh, PA 15260
deiters@pitt.edu

Table of Content

**Synthesis Protocols**	Synthesis of 4-amino-*N*-butyl-1,8-naphthalimide (ABNI)	2
	Synthesis of azido protected fluorophores	3
	Synthesis of phosphines	8
**Supporting Table 1**	Phosphorous NMR shifts of phosphines	10
**Supporting Table 2**	Data for pseudo first-order kinetic studies	10
**Supporting Figure 1**	Fluorophore activation of all azide/phosphine combinations	11
**Supporting Figures 2-11**	HPLC-MS analysis of reaction between azide and phosphine	12
**Supporting Figures 12-15**	pH dependence study of the reaction between azide and phosphine	20
**References**		22

Synthesis of 4-amino-*N*-butyl-1,8-naphthalimide (ABNI)

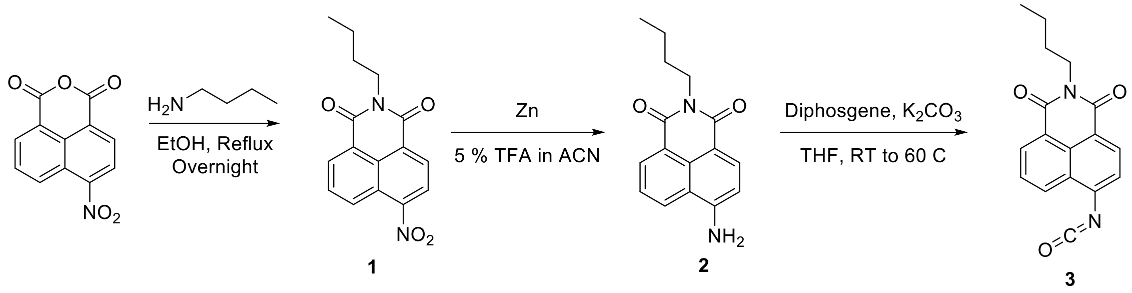

2-Butyl-6-nitro-1H-benz[de]isoquinoline-1,3(2H)-dione (1): 4-Nitro-1,8-napthalic anhydride (1.00 g, 4.1 mmol, 1 eq) was dissolved in ethanol (60 mL) and a solution of 1-butylamine (420 mg, 5.74 mmol, 1.4 eq) in ethanol (20 mL) and added. The mixture was stirred at reflux overnight and the volatiles (ethanol and butylamine) were removed under reduced pressure (3 hours). The crude mixture was purified via flash chromatography on silica gel, eluting with 15% EtOAc/hexanes, and **1** was obtained as an off-white solid (778 mg, 72%). ^{1}H NMR (400 MHz, $CDCl_3$) δ = 8.84 (d, 1H), 8.73 (d, 1H), 8.68 (d, 1H), 8.40 (d, 1H), 7.98 (t, 1H), 4.19 (t, 2H), 1.71 (m, 2H), 1.45 (m, 2H), 0.98 (t, 3H). ^{13}C NMR (100 MHz, $CDCl_3$) δ= 163.5, 162.7, 149.8, 132.6, 130.1, 130.0, 129.5, 129.3, 127.3, 124.1, 123.9, 123.3, 40.9, 30.3, 20.5, 14.0. HRMS-ESI: m/z calculated for $C_{16}H_{15}O_4N_2$ [M+H$^+$]: 299.10263; observed: 299.10256. The analytical data matches literature reports.[1]

6-Amino-2-butyl-1H-benz[de]isoquinoline-1,3(2H)-dione (2): The nitroarene **1** (60 mg, 20 mmol, 1 eq) was dissolved in 5 % TFA in acetonitrile (1:1, 6 mL), added to a nitrogen purged flask containing zinc powder (10 eq), and the resulting suspension was stirred at room temperature. The reaction was stirred under an inert atmosphere for 1 hour. The reaction mixture was filtered through celite and the filtrate concentrated under reduced pressure. The crude mixture was purified via flash chromatography eluting a stepwise gradient from DCM to 5% MeOH/DCM, and **2** was obtained as an orange solid. (54 mg, 100%). ^{1}H NMR (400 MHz, DMSO-d_6) δ = 8.61 (d, 1H), 8.42 (d, 1H), 8.19 (d, 1H), 7.65 (t, 1H), 7.44 (b, 2H), 6.84 (d, 1H), 4.01 (t, 2H), 1.58 (m, 2H), 1.33 (m, 2H), 0.91 (t, 3H). ^{13}C NMR (125 MHz, DMSO-d_6) δ= 163.4, 162.9, 153.0, 139.0, 137.3, 131.6, 130.9, 129.2, 128.3, 126.4, 119.3, 76.4, 29.6, 19.8, 13.7. HRMS-ESI: m/z calculated for $C_{16}H_{17}O_2N_2$ [M+H$^+$]: 269.12845; observed: 269.12665. The analytical data matches literature reports.[1]

2-Butyl-6-isocyanato-1H-benz[de]isoquinoline-1,3(2H)-dione (3): The aniline **2** (100 mg, 0.373 mmol, 1 eq) was dissolved in THF (1 mL) in a flame dried and nitrogen purged flask containing K_2CO_3 (154.8 mg, 1.22 mmol, 3 eq). Diphosgene (90 µL, 0.746 mmol, 2 eq) was added at room temperature and the reaction was sealed and stirred for 4 hours at 60° C. Volatiles were removed under reduced pressure (3 hours) to obtain the crude isocyanate **3** as a yellow-orange solid (110 mg, 100%). ^{1}H NMR (500 MHz, $CDCl_3$) δ = 8.65 (d, 1H), 8.54 (d, 1H), 8.44 (d, 1H), 7.82(t, 1H), 7.53 (d, 1H), 4.17 (t, 2H), 1.71 (m, 2H), 1.44 (m, 2H), 0.97 (t, 3H). ^{13}C NMR (125 MHz, $CDCl_3$) δ= 164.1, 163.6, 136.5, 132.3, 131.7, 129.3, 127.7, 123.4, 120.5, 40.5, 30.4, 20.6, 14.0. Due to the low stability of the isocyanate, an HRMS spectrum was not obtained.

Synthesis of azido-protected fluorophores

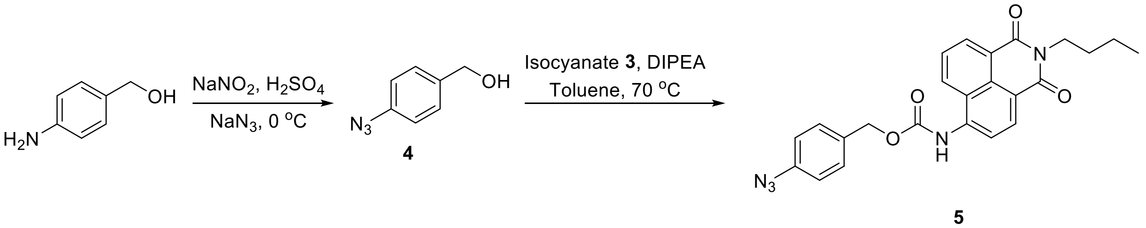

4-Azidobenzyl alcohol (4): Sodium nitrite (306 mg, 4.5 mmol, 1.125 eq.) in water was added to a solution of 4-aminobenzyl alcohol (500 mg, 4 mmol, 1 eq.) in 5 M HCl (16 mL) at 0℃. The mixture was stirred for 30 minutes and sodium azide (1.04 g, 16 mmol, 4 eq) was added in four portion over 1 hour with vigorous effervescence. The resulting mixture was stirred at 0 ˚C for 2 hours after which the reaction mixture was quenched with ice water (100 ml). The solution was basified to pH 8 by adding solid $NaHCO_3$ and was extracted with EtOAc (3 × 30 ml). The combined organic layer was washed with brine (100 ml), dried over $MgSO_4$, filtered, and concentrated. The crude the product was purified by flash column chromatography eluting 30% EtOAc/hexane affording **4** as a yellow solid (540.9 mg, 90%). ^{1}H NMR (300 MHz, $CDCl_3$) δ = 7.32-7.35 (d, 2H), 6.99-7.01 (d, 2H), 4.64-4.65 (d, 2H), 1.73 (b, 1H). ^{13}C NMR (100 MHz, $CDCl_3$) δ= 139.6, 137.8, 128.8, 119.3, 65.0. HRMS-ESI m/z calculated for $C_7H_7N_3$ [M+H-N_2-H_2O]: 104.03817; observed: 104.04948. The analytical data matches literature reports.[2]

4-Azidobenzyl 2-butyl-1,3-dioxo-2,3-dihydro-1H-benz[de]isoquinolin-6-ylcarbamate (5): The isocyanate **3** (50 mg, 0.170 mmol, 1 eq) was dissolved in toluene (1 mL). This solution was added to a flame dried flask containing the alcohol **4** (22.8 mg, 0.153 mmol, 0.9 eq). DIPEA (147 µL, 0.85 mmol, 5 eq) was added to the reaction mixture which was stirred for 12 hours at 70 ˚C. The reaction mixture was concentrated under reduced pressure and extracted using DCM (3 x 10 mL). The organic layers were combined, washed with brine (1 x 40 mL), dried over anhydrous sodium sulfate, filtered, and concentrated under reduced pressure. The crude mixture was purified via flash column chromatography eluting Hexanes to 20% EtOAc in Hexanes in a stepwise gradient yielding **5** as a light yellow solid (34.1 mg, 50 %). ^{1}H NMR (500 MHz, DMSO-d_6) δ = 10.39 (b, 1 H), 8.70 (m, 2H), 8.49 (d, 1H), 8.19 (d, 1H), 7.83 (t, 1H), 7.54 (d, 2H), 7.17 (d, 2H), 5.25 (s, 2H), 4.03 (t, 2H), 1.60 (m, 2H), 1.34 (m, 2H), 0.92 (t, 3H). ^{13}C NMR (125 MHz, DMSO-d_6) δ= 163.4, 162.9, 153.9, 140.6, 139.3, 133.1, 131.7, 130.9, 130.2, 129.3, 128.3, 126.4, 123.9, 122.2, 119.2, 118.2, 117.1, 66.0, 29.7, 19.8, 13.7. HRMS-ESI: m/z calculated for $C_{24}H_{22}O_4N_5$ [M+H]: 444.16663; observed: 444.16552.

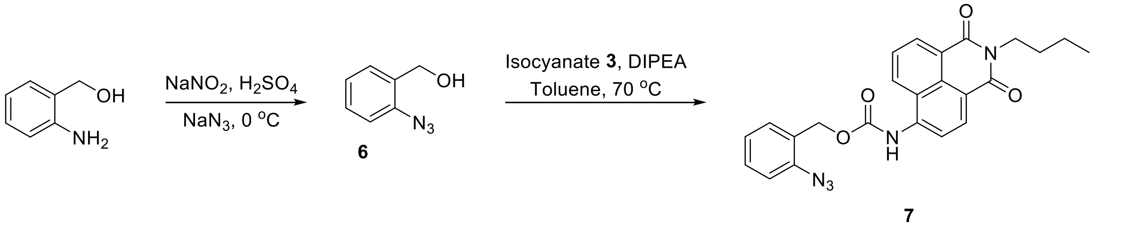

2-Azidobenzyl alcohol (6): Sodium nitrite (346.7 mg, 5.02 mmol, 1.125 eq.) in water (6 mL) was added to a solution of 2-aminobenzyl alcohol (550 mg, 4.46 mmol, 1 eq.) in 5 M HCl (18 mL) at 0℃. The mixture was stirred for 30 minutes and sodium azide (1.16 g, 17.8 mmol, 4 eq) was added in four portion over 1 hour with vigorous effervescence. The resulting mixture was stirred at 0 C for 2 hours after which the reaction mixture was quenched with ice water (100 ml). The solution was basified to pH 8 by adding solid $NaHCO_3$ and was extracted with EtOAc (3 × 30 ml). The combined organic layer was washed with brine (100 ml), dried over $MgSO_4$, filtered, and concentrated. The crude the product was purified by flash column chromatography eluting 30% EtOAc/hexane affording **6** as a yellow solid (651.8, 100%). ^{1}H NMR (500 MHz, $CDCl_3$) δ = 7.35 (m, 2H), 7.15 (m, 2H), 4.64 (s, 2H), 2.06 (t, 1H). ^{13}C NMR (125 MHz, $CDCl_3$) δ = 138.1, 132.1, 129.5, 129.3, 118.2, 61.9. HRMS-ESI: m/z calculated for C_7H_6N [M+H-N_2-H_2O]: 104.04948; observed 104.04946. The analytical data matches literature reports.[3]

2-Azidobenzyl 2-butyl-1,3-dioxo-2,3-dihydro-1H-benz[de]isoquinolin-6-ylcarbamate (7): The isocyanate **3** (50 mg, 0.170 mmol, 1 eq) was dissolved in Toluene (1 mL). This solution was added to a flame dried flask containing the alcohol **6** (23 mg, 0.153 mmol, 0.9 eq). DIPEA (147 µL, 0.85 mmol, 5 eq) was added to the

reaction mixture which was stirred for 12 hours at 70 ˚C. The reaction mixture was concentrated under reduced pressure and extracted using DCM (3 x 10 mL). The organic layers were combined, washed with brine (1 x 40 mL), dried over anhydrous sodium sulfate, filtered, and concentrated under reduced pressure. The crude mixture was purified via flash column chromatography eluting Hexanes to 20% EtOAc in Hexanes in a stepwise gradient yielding **7** as a light yellow solid (34.1 mg, 50 %). ^{1}H NMR (500 MHz, DMSO-d_6) δ = 10.40 (b, 1H), 8.71 (d, 1H), 8.49 (m, 2H), 8.20 (d, 1H), 7.82 (t, 1H), 7.58 (d, 1H), 7.49 (t, 1H), 7.39 (d, 1H), 7.26 (t, 1H), 5.21 (s, 2H), 4.03 (t, 2H), 1.61 (m, 2H), 1.35 (m, 2H), 0.94 (t, 3H). ^{13}C NMR (125 MHz, DMSO-d_6) δ = 163.5, 162.9, 153.9, 131.7, 130.9, 130.4, 130.1, 129.3, 128.3, 126.9, 126.3, 125.0, 122.2, 118.8, 118.2, 62.3, 29.7, 19.8, 13.7. HRMS-ESI: m/z calculated for $C_{24}H_{22}O_4N_5$ [M+H]: 444.16663; observed: 444.16578.

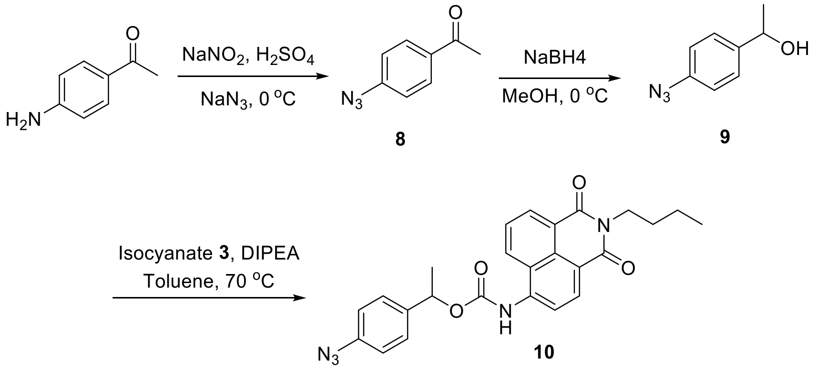

4-Azidoacetophenone (8): Sodium nitrite (287 mg, 4.16 mmol, 1.125 eq.) in water (5 mL) was added to a solution of 4-aminoacetophenone(500 mg, 3.7 mmol, 1 eq.) in 5 M HCl (15 mL) at 0℃. The mixture was stirred for 30 minutes and sodium azide (490 mg, 7.54 mmol, 8eq) was added in four portion over 1 hour with vigorous effervescence. The resulting mixture was stirred at 0 C for 2 hours after which the reaction mixture was quenched with ice water (100 ml). The solution was basified to pH 8 by adding solid NaHCO3 and was extracted with EtOAc (3 × 30 ml). The combined organic layer was washed with brine (100 ml), dried over $MgSO_4$, filtered, and concentrated. The crude the product was purified by flash column chromatography eluting 30% EtOAc/hexane affording **8** as a yellow solid (591.5 mg, 100%). ^{1}H NMR (300 MHz, $CDCl_3$) δ = 7.96 (d, 2H), 7.08 (d, 2H), 2.58 (s, 3H). ^{13}C NMR (100 MHz, $CDCl_3$) δ = 196.6, 144.9, 133.9, 130.3, 119.0, 26.5. HRMS-ESI: m/z calculated for $C_8H_8ON_3$ [M+H]: 162.06619; observed: 162.06631. The analytical data matches literature reports.[4]

1-(4-Azidophenyl)ethan-1-ol (9): Sodium borohydride (11.7 mg, .311 mmol, 0.5 eq) was weighed into a flame dried flask. Compound **8** (100 mg, 0.621 mmol, 1 eq) was dissolved in anhydrous MeOH and added under an inert atmosphere at 0 ˚C. The reaction was warmed to room temperature until completion verified by TLC. The reaction mixture was concentrated under reduced pressure and quenched with water. The product was extracted using EtOAc (3 x 25 mL) and was washed with Brine (1 x 25 mL), dried with anhydrous $MgSO_4$, filtered, and concentrated. The crude product was purified by flash column chromatography eluting 30 % EtOAc in Hexanes to yield **9** (76 mg, 75 %). ^{1}H NMR (300 MHz, $CDCl_3$) δ = 7.34 (d, 2H), 6.99 (d, 2H), 4.87 (m, 1H), 1.79 (b, 1H), 1.46 (d, 3H). ^{13}C NMR (100 MHz, $CDCl_3$) δ = 142.7, 139.3, 127.1, 119.2, 70.0, 25.4. HRMS-ESI: m/z calculated for $C_8H_{10}ON$ [M+H-N_2]: 136.07569; observed: 136.07572.

1-(4-Azidobenzen-2-yl)ethyl 2-butyl-1,3-dioxo-2,3-dihydro-1H-benz[de]isoquinolin-6-ylcarbamate (10): The isocyanate **3** (50 mg, 0.170 mmol, 1 eq) was dissolved in toluene (1 mL). This solution was added to a flame-dried flask containing the alcohol **9** (40.7 mg, 0.153 mmol, 0.9 eq). DIPEA (147 µL, 0.85 mmol, 5 eq) was added to the reaction mixture which was stirred for 12 hours at 70 ˚C. The reaction mixture was concentrated under reduced pressure and extracted using DCM (3 x 10 mL). The organic layers were combined, washed with brine (1 x 40 mL), dried over anhydrous sodium sulfate, filtered, and concentrated under reduced pressure. The crude mixture was purified via flash column chromatography eluting Hexanes to 20% EtOAc in Hexanes in a stepwise gradient yielding **10** as a light yellow solid (36.8 mg, 43 %). ^{1}H NMR (500 MHz, DMSO-d_6) δ = 10.36 (b, 1H), 8.70 (d, 1H), 8.50 (d, 1H), 8.45 (d, 1H), 8.14 (d, 1H), 7.83 (t, 1H), 7.53 (d, 2H), 7.16 (d, 2H), 5.91 (q, 2H), 4.03 (t, 2H), 1.60 (m, 5H), 1.34 (m, 2H), 0.93 (t, 3H). ^{13}C NMR (125 MHz, DMSO) δ = 163.4, 162.9, 153.3, 140.6, 138.8, 138.7, 129.3, 128.3, 127.7, 126.3, 123.8, 122.2, 119.7, 118.2, 117.0, 72.5, 29.6, 22.1, 19.8, 13.7. HRMS-ESI: m/z calculated for $C_{25}H_{24}O_4N_5$ [M+H]: 458.18228; observed: 458.18071.

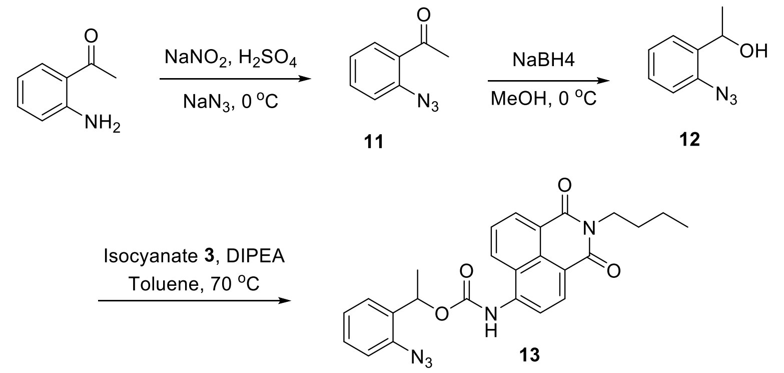

2-Azidoacetophenone (11): Sodium nitrite (287 mg, 4.16 mmol, 1.125 eq.) in water was added to a solution of 2-aminoacetophenone (500 mg, 3.7 mmol, 1 eq.) in 5 M HCl (16 mL) at 0℃. The mixture was stirred for 30 minutes and sodium azide (962 mg, 14.8 mmol, 4 eq) was added in four portion over 1 hour with vigorous effervescence. The resulting mixture was stirred at 0 C for 2 hours after which the reaction mixture was quenched with ice water (100 ml). The solution was basified to pH 8 by adding solid $NaHCO_3$ and was extracted with EtOAc (3 × 30 ml). The combined organic layer was washed with brine (100 ml), dried over $MgSO_4$, filtered, and concentrated. The crude the product was purified by flash column chromatography eluting 30% EtOAc/hexane affording **11** as a yellow solid (578 mg, 98%). ^1H NMR (400 MHz, $CDCl_3$) δ = 7.71 (d, 1H), 7.52 (t, 1H), 7.21 (m, 2H), 2.64 (s, 3H). ^{13}C NMR (100 MHz, $CDCl_3$) δ = 199.4, 138.9, 133.31, 133.3, 130.6, 125.0, 119.4, 31.4. HRMS-ESI: m/z calculated for C_8H_8ON [M+H-N_2]: 134.06004; observed: 134.05972. The analytical data matches literature reports.[5]

1-(2-Azidophenyl)ethan-1-ol (12): Sodium borohydride (23.1 mg, 0.61 mmol, 0.5 eq) was weighed into a flame dried flask. **11** (197 mg, 1.22 mmol,1 eq) was dissolved in anhydrous MeOH and added under an inert atmosphere at 0 ˚C. The reaction was warmed to room temperature until completion verified by TLC. The reaction mixture was concentrated under reduced pressure and quenched with water. The product was extracted using EtOAc (3 x 25 mL) and was washed with Brine (1 x 25 mL), dried with anhydrous $MgSO_4$, filtered, and concentrated. The crude product was purified by flash column chromatography eluting 30 % EtOAc in Hexanes to yield **12** (185.7 mg, 93%). ^1H NMR (400 MHz, CDCl3) δ = 7.46 (d, 1H), 7.31 (m, 1H), 7.15 (m, 2H), 5.07 (q, 1H), 2.24 (b, 1H),1.46 (d, 3H). ^{13}C NMR (100 MHz, $CDCl_3$) δ = 136.9, 136.7, 128.7, 126.7, 125.3, 118.2, 66.2, 23.8. HRMS-ESI: m/z calculated for $C_8H_{10}ON$ [M+H-N_2]: 136.07569; observed: 136.07547.

1-(2-Azidobenzen-2-yl)ethyl 2-butyl-1,3-dioxo-2,3-dihydro-1H-benz[de]isoquinolin-6-ylcarbamate (13): The isocyanate **3** (50 mg, 0.170 mmol, 1 eq) was dissolved in toluene (1 mL). This solution was added to a flame-dried flask containing the alcohol **12** (25.0 mg, 0.153 mmol, 0.9 eq). DIPEA (147 µL, 0.85 mmol, 5 eq) was added to the reaction mixture that was stirred for 12 hours at 70 ˚C. The reaction mixture was concentrated under reduced pressure and extracted using DCM (3 x 10 mL). The organic layers were combined, washed with brine (1 x 40 mL), dried over anhydrous sodium sulfate, filtered, and concentrated under reduced pressure. The crude mixture was purified via flash column chromatography eluting hexanes to 20% EtOAc in hexanes in a stepwise gradient yielding **13** as a light yellow solid (28.1 mg, 41 %). ^1H NMR (500 MHz, DMSO-d_6) δ = 10.39 (b, 1H), 8.72 (d, 1H), 8.51 (d, 1H), 8.45 (d, 1H), 8.14 (d, 1H), 7.85 (t, 1H), 7.59 (d, 1H), 7.43 (t, 1H), 7.35 (d,1H), 7.27 (t, 1H), 6.05 (q, 1H), 4.03 (t, 2H), 1.59 (m, 5H), 1.34 (m, 2H), 0.92 (t, 3H). ^{13}C NMR (125 MHz, DMSO-d_6) δ = 163.8, 163.2, 153.5, 140.9, 136.4, 133.3, 132.0, 131.2, 129.6, 128.6, 126.7, 126.5, 125.7, 124.2, 122.6, 119.0, 118.6, 117.4, 68.6, 30.00, 21.7, 20.1,14.0. HRMS-ESI: m/z calculated for $C_{25}H_{24}O_4N_5$ [M+H]: 458.18228; observed: 458.18105.

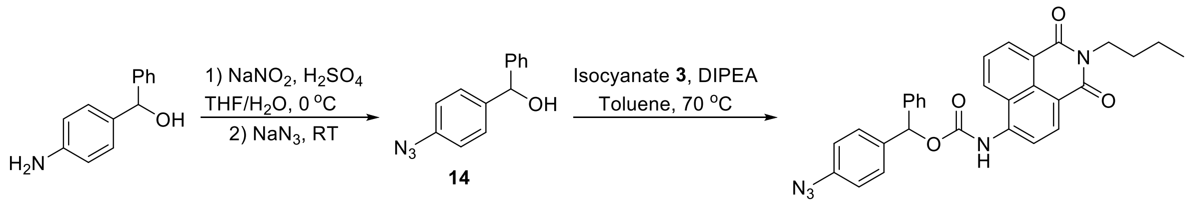

4-Azidobenzhydrol (14): 4-Aminobenzyhydrol (890 mg, 4.47 mmol) was dissolved in THF (6.4 mL)/H_2O (19 mL) and concentrated sulfuric acid (1.5 mL) and $NaNO_2$ (370 mg, 5.36 mmol) in H_2O (3.1 mL) were added at 0 ˚C. The reaction mixture was stirred for 1 h at room temperature and NaN_3 (348 mg, 5.36 mmol) was added as a solution in H_2O (1.5 mL). The reaction mixture was stirred for 1 h at room temperature and extracted with CH_2Cl_2. The organic layer was washed with and brine and dried over Na_2SO_4. The solvent was removed in vacuo and the residue was purified by flash chromatography on silica gel, eluting with hexanes/EtOAc (9:1 to 4:1) to give compound **14** (887 mg, 88%) as a yellow oil. ^{1}H NMR (400 MHz, $CDCl_3$) δ= 7.32 (m, 7H), 7.00 (d, 2H), 5.83 (s 1H). ^{13}C NMR (100 MHz, $CDCl_3$) δ= 143.5, 140.5, 139.1, 128.5, 127.9, 127.7, 126.4, 119.0, 75.6. HRMS-ESI: m/z calculated for $C_{13}H_{12}ON$ [M+H-N2]: 198.0913; observed: 198.0972.

1-(4-Azidobenzen-2-yl)benzyl 2-butyl-1,3-dioxo-2,3-dihydro-1H-benz[de]isoquinolin-6-ylcarbamate (15): The isocyanate **3** (50 mg, 0.170 mmol, 1 eq) was dissolved in toluene (1 mL). This solution was added to a flame dried flask containing the alcohol **14** (34.5 mg, 0.153 mmol, 0.9 eq). DIPEA (147 µL, 0.85 mmol, 5 eq) was added to the reaction mixture which was stirred for 12 hours at 70 ˚C. The reaction mixture was concentrated under reduced pressure and extracted using DCM (3 x 10 mL). The organic layers were combined, washed with brine (1 x 40 mL), dried over anhydrous sodium sulfate, filtered, and concentrated under reduced pressure. The crude mixture was purified via flash column chromatography eluting hexanes to 20% EtOAc in hexanes in a stepwise gradient yielding **15** as a light yellow solid (40.5 mg, 51 %). ^{1}H NMR (400 MHz, $CDCl_3$) δ= 8.62 (d, 1H), 8.57 (d, 1H), 8.35 (d, 1H), 8.19 (d, 1H), 7.75 (t, 1H), 7.61 (s, 1H), 7.37 (m, 7H), 7.03 (d, 1H), 6.96 (s, 1H), 4.16 (t, 1H), 1.71 (m, 2H), 1.44 (m, 2H), 0.97 (t, 3H). ^{13}C NMR (100 MHz, $CDCl_3$) δ= 164.1, 163.6, 152.3, 140.1, 139.2, 138.6, 136.2, 132.4, 131.2, 128.8, 128.5, 127.1, 126.7, 125.8, 123.5, 122.9, 119.3, 118.1, 116.9, 78.4, 40.2, 30.2, 29.7, 20.4, 13.8. HRMS-ESI: m/z calculated for $C_{30}H_{26}O_5N_4$ [M+H]: 520.1979; observed: 520.1978.

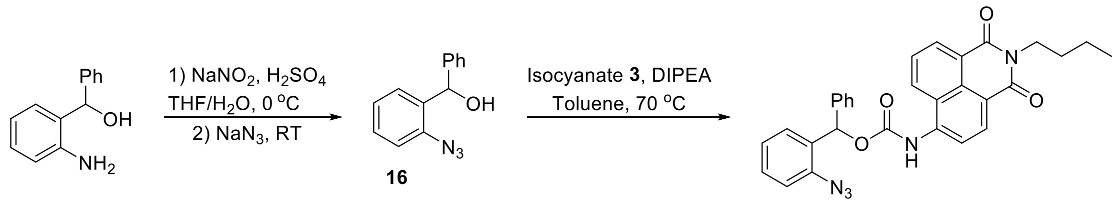

2-Azidobenzhydrol (16): Compound 1 (50 mg, 0.251 mmol) was dissolved in THF (0.5 mL)/H_2O (1.6 mL) and cH_2SO_4 (75 µL) and $NaNO_2$ (21 mg, 0.301 mmol) in H_2O (0.2 mL) were added at 0 ˚C. The reaction mixture was stirred for 1 h at room temperature and NaN_3 (20 mg, 0.301 mmol) was added as a solution in H_2O (0.2 mL). The reaction mixture was stirred for 15 h at room temperature and extracted with CH_2Cl_2. The organic layer was washed with and brine and dried over Na_2SO_4. The solvent was removed in vacuo and the residue was purified by flash chromatography on silica gel, eluting with hexanes/EtOAc (9:1) to give compound **16** (53 mg, 94%) as a yellow oil. ^{1}H NMR (400 MHz, $CDCl_3$) δ= 7.47 (dd, 1H), 7.27 (m, 8H), 6.04 (d, 1H), 2.53 (d, 1H). ^{13}C NMR (100 MHz, $CDCl_3$) δ= 142.8, 137.0, 134.7, 128.8, 128.4, 128.0, 127.6, 126.6, 125.0, 118.2, 71.6. HRMS-ESI: m/z calculated for $C_{13}H_{12}ON$ [M+H-N2]: 198.0913; observed: 198.0906. The analytical data matches literature reports.[6]

1-(2-Azidobenzen-2-yl)benzyl 2-butyl-1,3-dioxo-2,3-dihydro-1H-benz[de]isoquinolin-6-ylcarbamate (17): The isocyanate **3** (50 mg, 0.170 mmol, 1 eq) was dissolved in toluene (1 mL). This solution was added to a flame dried flask containing the alcohol **16** (34.5 mg, 0.153 mmol, 0.9 eq). DIPEA (147 µL, 0.85 mmol, 5 eq) was added to the reaction mixture which was stirred for 12 hours at 70 ˚C. The reaction mixture was concentrated under reduced pressure and extracted using DCM (3 x 10 mL). The organic layers were

combined, washed with brine (1 x 40 mL), dried over anhydrous sodium sulfate, filtered, and concentrated under reduced pressure. The crude mixture was purified via flash column chromatography eluting with hexanes to 20% EtOAc in hexanes in a stepwise gradient yielding **17** as a light yellow solid (50.1 mg, 63 %). ^{1}H NMR (400 MHz, $CDCl_3$) δ= 8.63 (d, 1H), 8.58 (d, 1H), 8.38 (d, 1H), 8.19 (d, 1H), 7.77 (t, 1H), 7.55 (s, 1H), 7.49 (d, 1H), 7.39 (m, 6H), 7.20 (m, 3H), 4.17 (t, 2H), 1.71 (m, 2H), 1.45 (m, 2H), 0.97 (t, 3H). ^{13}C NMR (100 MHz, $CDCl_3$) δ= 164.1, 163.6, 152.2, 138.7, 138.6, 137.5, 132.4, 131.2, 130.7, 129.5, 128.8, 128.6, 128.4, 127.6, 127.3, 126.6, 125.8, 124.9, 123.5, 122.9, 118.4, 118.0, 116.9, 74.1, 40.2, 30.2, 29.7, 13.8. HRMS-ESI: m/z calculated for $C_{30}H_{26}O_5N_4$ [M+H]: 520.1979; observed: 520.1971.

Synthesis of phosphines

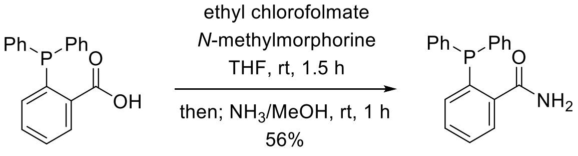

21 22

2-(Diphenylphosphino)benzamide (22): 2-(Diphenylphosphino)benzoic acid **21** (500 mg, 1.63 mmol) was dissolved in THF (5.0 mL) and *N*-methylmorphorine (0.18 mL, 1.63 mmol) and ethyl chloroformate (0.16 mL, 1.63 mmol) were added. The reaction mixture was stirred at rt for 1.5 h. 7 M NH_3/MeOH (3.5 mL, 24.5 mmol) was added and the reaction mixture was stirred for 1 h at rt. The solvent was removed in vacuo and the resulting residue was purified by flash chromatography on silica gel, eluting with hexanes/EtOAc (1:1) to give compound **22** (280 mg, 56%) as a white solid. ^{1}H NMR (400 MHz, $CDCl_3$) δ= 7.69 (ddd, 1H), 7.42-7.27 (m, 12H), 6.99 (ddd, 1H), 6.03 (brs, 1H), 5.64 (brs, 1H). ^{13}C NMR (101 MHz, $CDCl_3$) δ= 170.5, 139.8, 139.7, 136.8, 136.7, 136.4, 136.2, 134.3, 134.0, 133.8, 130.6, 128.9, 128.8, 128.6, 128.5, 128.2, 128.1. ^{31}P NMR (162 MHz, $CDCl_3$) δ= -8.71. HRMS-ESI: m/z calculated for $C_{19}H_{16}ONP$ [M+H] 306.3242; observed 306.1042. The analytical data matches literature reports.[7]

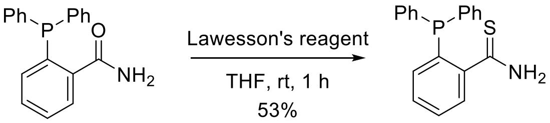

22 23

2-(Diphenylphosphino)benzthioamide (23): Compound **22** (31 mg, 0.100 mmol) was dissolved in THF (1.5 mL) and Lawesson's reagent (49 mg, 0.120 mmol) was added. The reaction mixture was stirred at rt for 1 h. The solvent was removed in vacuo and the resulting residue was dissolved into AcOEt and washed with sat. $NaHCO_3$ aq. and brine and dried over Na_2SO_4. The solvent was removed in vacuo and the resulting residue was purified by flash chromatography on silica gel, eluting with hexanes/EtOAc (4:1) to give compound **23** (17 mg, 53%) as a yellow solid. ^{1}H NMR (400 MHz, $CDCl_3$) δ= 7.66 (ddd, 1H), 7.61 (brs, 1H), 7.39-7.28 (m, 12H), 6.96 (ddd, 1H), 6.93 (brs, 1H). ^{13}C NMR (101 MHz, $CDCl_3$) δ= 204.6, 147.8, 147.5, 136.2, 136.1, 134.2, 133.9, 133.7, 132.5,132.4,132.1, 129.7, 129.2, 128.9, 128.8, 128.7, 128.4, 128.3. ^{31}P NMR (162 MHz, $CDCl_3$) δ= -12.80. HRMS-ESI: m/z calculated for $C_{19}H_{16}NPS$ [M+H] 322.0814; observed 322.0783.

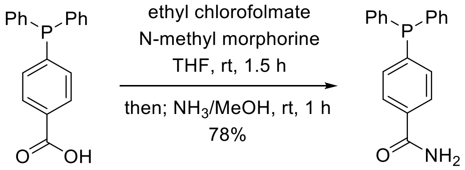

24 25

4-(Diphenylphosphino)benzamide (25): 4-(Diphenylphosphino)benzoic acid **24** (100 mg, 0.326 mmol) was dissolved in THF (5.0 mL) and *N*-methylmorphorine (40 μL, 0.359 mmol) and ethyl chloroformate (34 μL, 0.359 mmol) were added. The reaction mixture was stirred at rt for 1 h. 7 M NH_3/MeOH (0.7 mL, 4.89 mmol) was added and the reaction mixture was stirred for 1 h at rt. The solvent was removed in vacuo and the resulting residue was purified by flash chromatography on silica gel, eluting with hexanes/EtOAc (1:1) to give compound **25** (78 mg, 78%) as a white solid. ^{1}H NMR (400 MHz, $CDCl_3$) δ= 7.75 (dd, 2H), 7.37-7.31 (m, 12 H), 6.06 (brs, 1H), 5.68 (brs, 1H). ^{13}C NMR (101 MHz, $CDCl_3$) δ= 143.0, 142.9, 136.2, 136.1, 134.0, 133.8, 133.6, 133.4, 133.0, 129.1, 128.7, 128.6, 127.2, 127.1. ^{31}P NMR (162 MHz, $CDCl_3$) δ= -4.82. HRMS-ESI: m/z calculated for $C_{19}H_{16}ONP$ [M+H] 306.1042; observed 306.1041. The analytical data matches literature reports.[8]

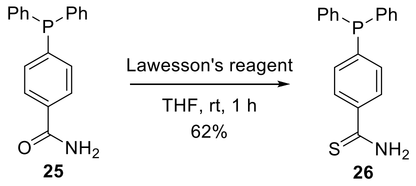

4-(Diphenylphosphino)benzthioamide (26): Compound **25** (100 mg, 0.328 mmol) was dissolved in THF (3.3 mL) and Lawesson's reagent (159 mg, 0.394 mmol) was added. The reaction mixture was stirred at rt for 1 h. The solvent was removed in vacuo and the resulting residue was dissolved into AcOEt and washed with sat. $NaHCO_3$ aq. and brine and dried over Na_2SO_4. The solvent was removed in vacuo and the resulting residue was purified by flash chromatography on silica gel, eluting with hexanes/EtOAc (4:1) to give compound **26** (65 mg, 62%) as a yellow solid. ^{1}H NMR (400 MHz, $CDCl_3$) δ= 7.81 (d, 1H), 7.38-7.28 (m, 12H). ^{13}C NMR (101 MHz, $CDCl_3$) δ= 202.2, 143.1, 143.0, 138.8, 136.1, 136.0, 134.0, 133.8, 133.4,133.2, 129.2, 128.7, 128.6, 126.7, 126.6. ^{31}P NMR (162 MHz, $CDCl_3$) δ= -4.75. HRMS-ESI: m/z calculated for $C_{19}H_{16}NPS$ [M+H] 322.0814; observed 322.0804.

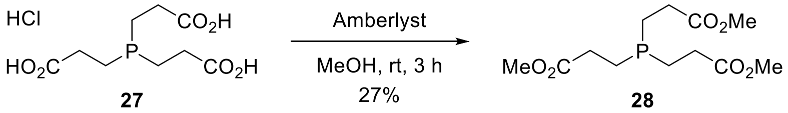

Tris(2-methoxycarbonylethyl)phosphine (28): Tris(2-carboxyethyl)phosphine hydrochloride **27** (250 mg, 0.874 mmol) was dissolved in MeOH (8.7 mL) and Amberlyst (250 mg) was added. The reaction mixture was stirred at rt for 3 h and filtrated. The reaction mixture was separated with CH_2Cl_2 and H_2O and organic layer was washed with brine and dried over Na_2SO_4. The solvent was removed in vacuo and the resulting residue was purified by flash chromatography on silica gel, eluting with CH_2Cl_2/MeOH (19:1) to give compound **28** (70 mg, 27%) as a colorless oil. ^{1}H NMR (400 MHz, $CDCl_3$) δ= 3.67 (s, 9H), 2.43 (dt, 6H), 1.72 (t, 6H). ^{13}C NMR (101 MHz, $CDCl_3$) δ= 173.6, 51.8, 30.4, 30.2, 21.5, 21.3. ^{31}P NMR (162 MHz, $CDCl_3$) δ= -25.11. HRMS-ESI: m/z calculated for $C_{12}H_{21}O_6P$ [M+H] 293.1149; observed 293.1141.

**Table S1:** Phosphorous NMR shifts of phosphines	
Phosphine	P^{31} NMR Shift
**8**	-25.75
**9**	-11.12
**10**	-5.92
**11**	-5.46
**12**	-4.82
**13**	-4.75
**14**	-4.59
**15**	-8.71
**16**	-12.8

*All NMR taken in $CDCl_3$ using phosphoric acid as a standard for a shift of 0.00 ppm.

Table S2: Data for pseudo first-order kinetic studies

	100 uM phosphine **15**
[**3**] (uM)	Rate (uM/min)
10	3.13E-01 ± 4.08E-03
1	5.73E-02 ± 1.36E-03
0.1	6.43E-03 ± 2.34E-04
	100 uM phosphine **15**
[**4**] (uM)	Rate (uM/min)
10	3.74E-01 ± 1.93E-02
1	6.78E-02 ± 3.01E-03
0.1	6.40E-03 ± 6.45E-04
	100 uM phosphine **9**
[**3**] (uM)	Rate (uM/min)
10	5.38E-02 ± 1.99E-03
1	1.30E-02 ± 1.35E-03
0.1	2.75E-03 ± 6.76E-05

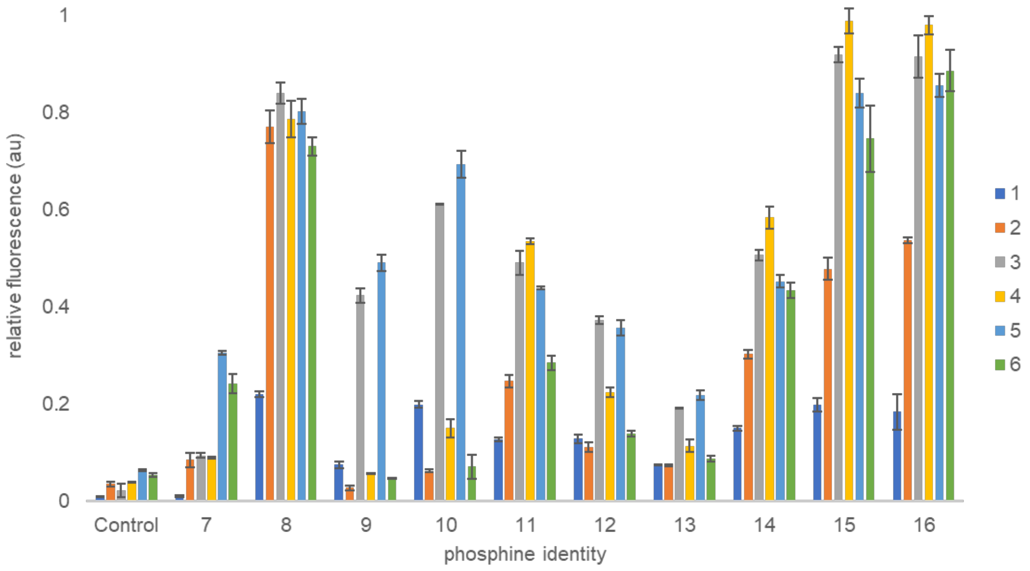

Figure S1. Azides **1-6** (10 μM) was treated with phosphines **7-16** (100 μM) in 20% DMSO in PBS (pH7.4) buffer. All possible azide and phosphine combinations were tested in triplicate (error bars represent standard deviation).

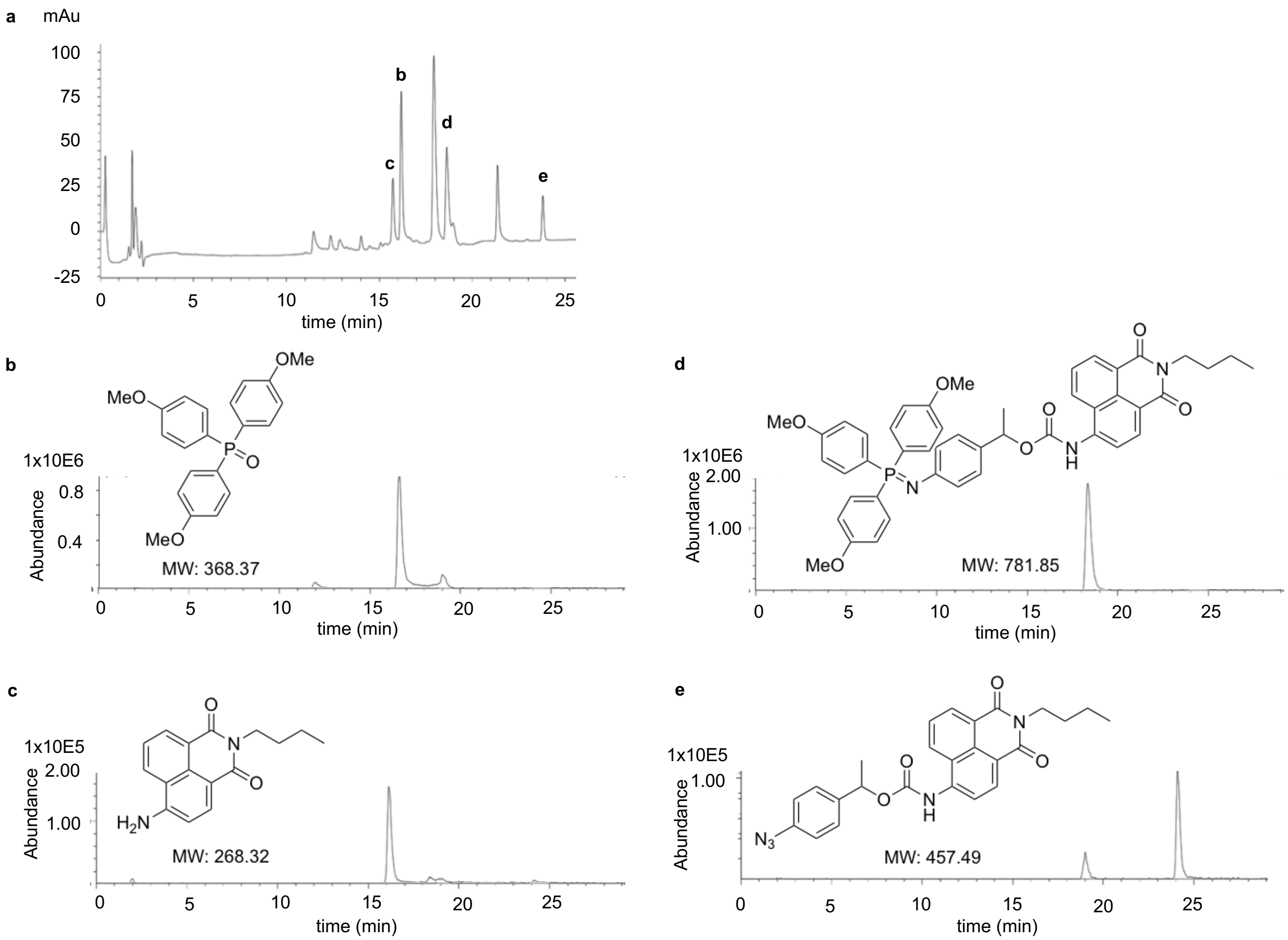

Figure S2. LC-MS analysis of reaction between azide **3** and phosphine **9.** (**a**) UV trace of reaction mixture with peaks labeled for reactants and intermediates indicated in mass traces. (**b**) Extracted LC-ESI-MS chromatogram for oxide of phosphine **9** (**c**) ABNI (**d**) aza-ylide formed by reaction of azide **3** and phosphine **9** (**e**) azide **3**.

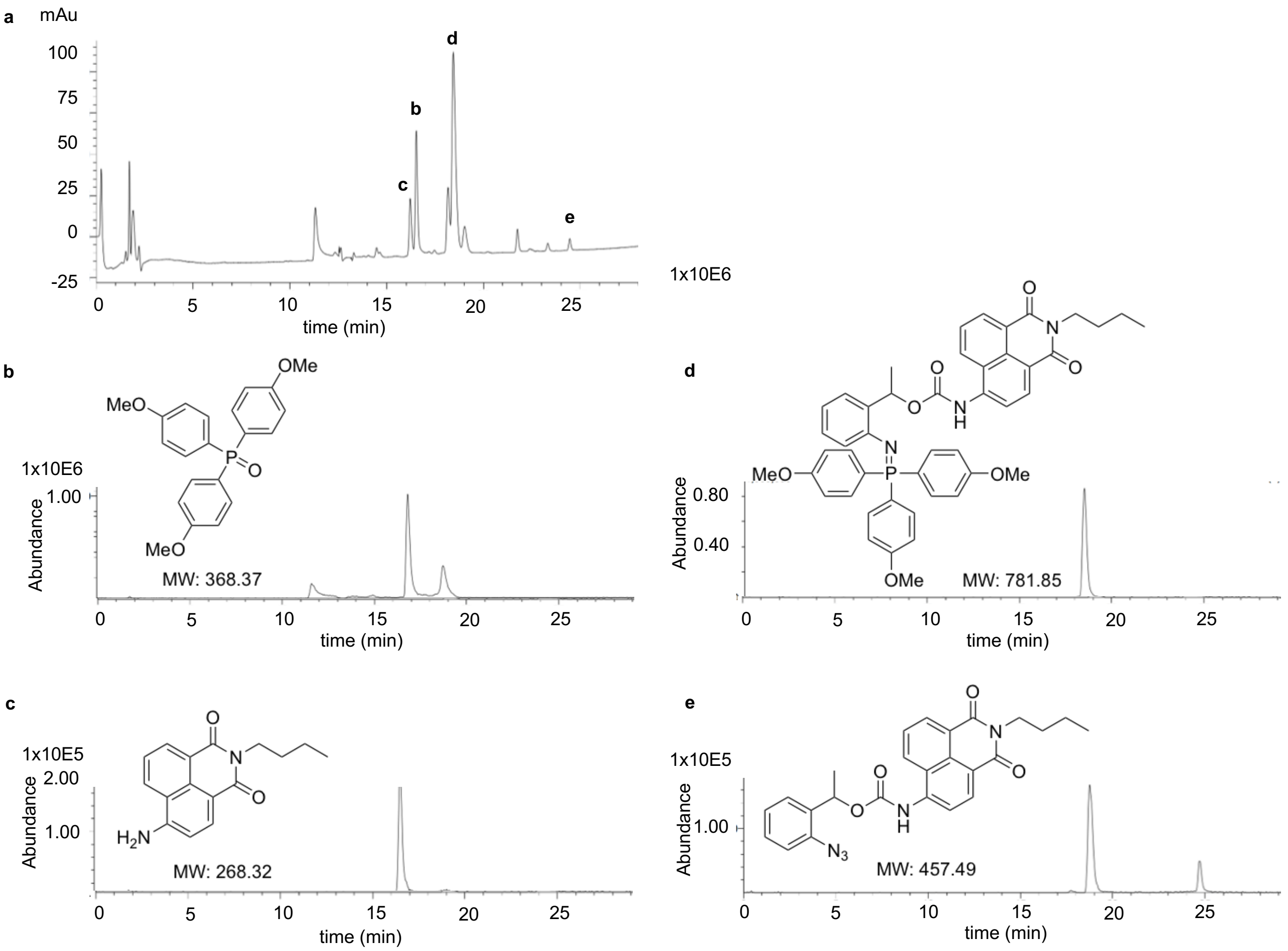

Figure S3. LC-MS analysis of reaction between azide **4** and phosphine **9.** (**a**) UV trace of reaction mixture with peaks labeled for reactants and intermediates indicated in mass traces. (**b**) Extracted LC-ESI-MS chromatogram for oxide of phosphine **9** (**c**) ABNI (**d**) aza-ylide formed by reaction of azide **4** and phosphine **9** (**e**) azide **4**.

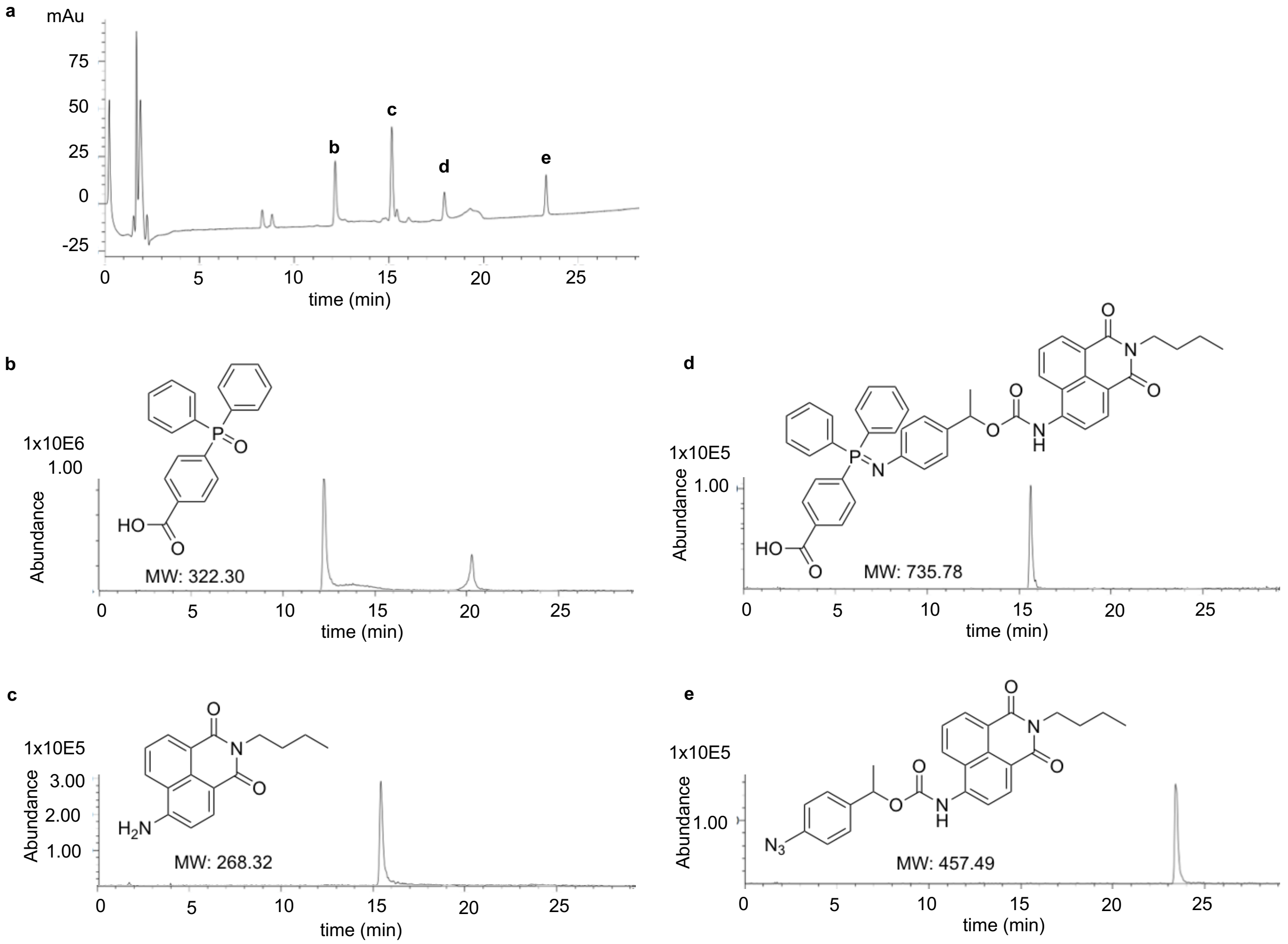

Figure S4. LC-MS analysis of reaction between azide **3** and phosphine **11.** (**a**) UV trace of reaction mixture with peaks labeled for reactants and intermediates indicated in mass traces. (**b**) Extracted LC-ESI-MS chromatogram for oxide of phosphine **11** (**c**) ABNI (**d**) aza-ylide formed by reaction of azide **3** and phosphine **11** (**e**) azide **3**.

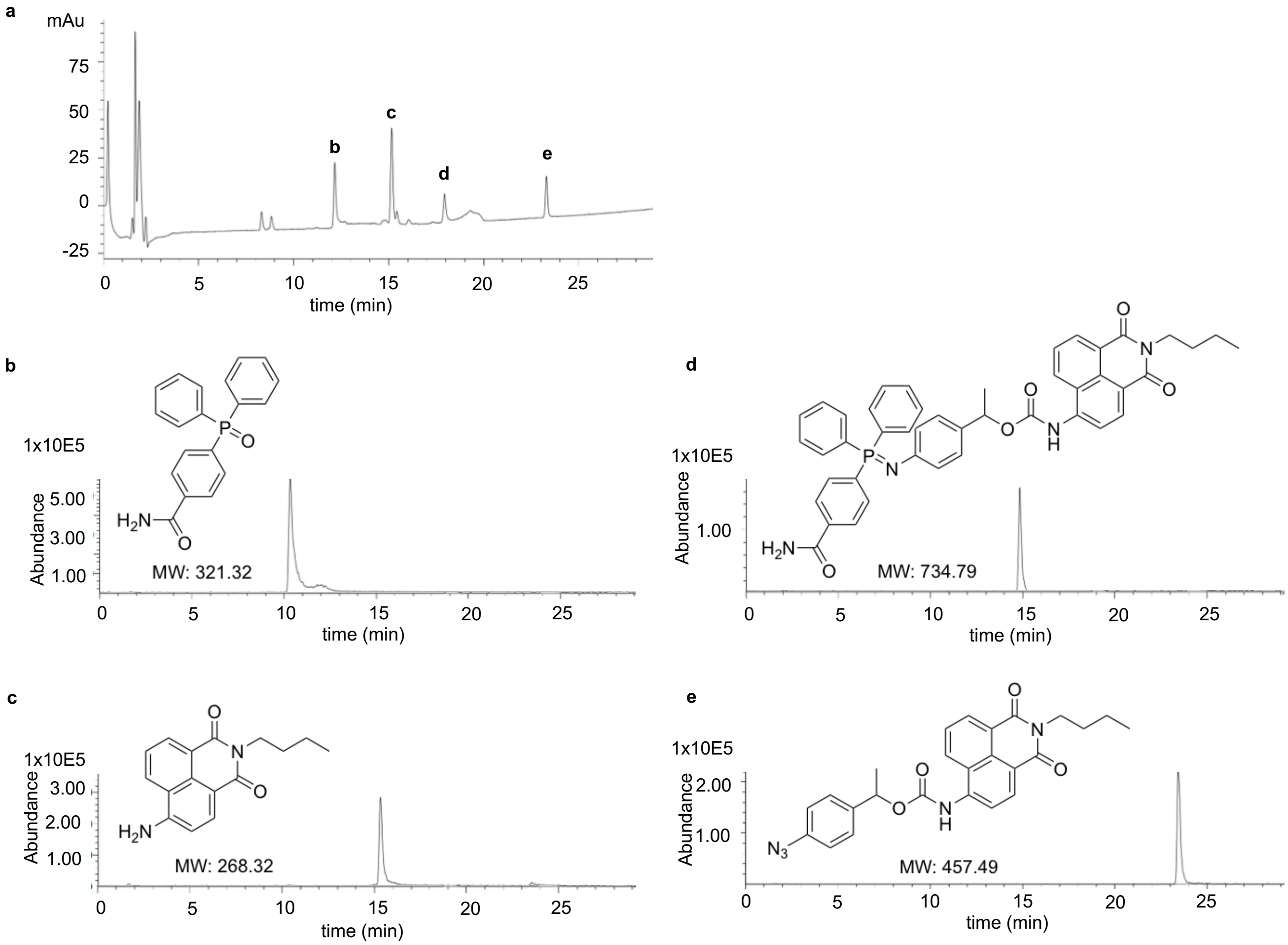

Figure S5. LC-MS analysis of reaction between azide **3** and phosphine **12.** (**a**) UV trace of reaction mixture with peaks labeled for reactants and intermediates indicated in mass traces. (**b**) Extracted LC-ESI-MS chromatogram for oxide of phosphine **12** (**c**) ABNI (**d**) aza-ylide formed by reaction of azide **3** and phosphine **12** (**e**) azide **3**.

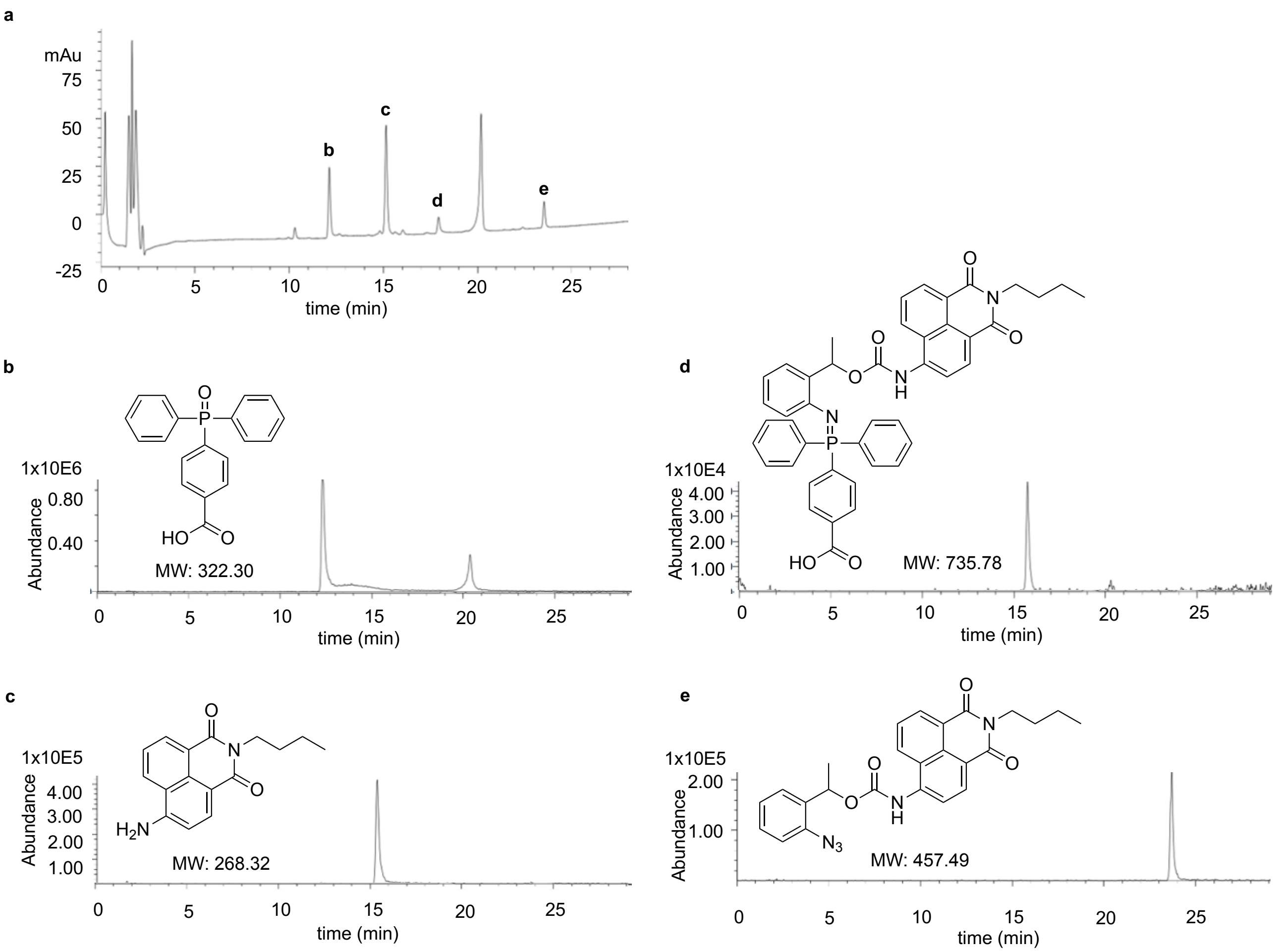

Figure S6. LC-MS analysis of reaction between azide **4** and phosphine **11.** (**a**) UV trace of reaction mixture with peaks labeled for reactants and intermediates indicated in mass traces. (**b**) Extracted LC-ESI-MS chromatogram for oxide of phosphine **11** (**c**) ABNI (**d**) aza-ylide formed by reaction of azide **4** and phosphine **11** (**e**) azide **4**.

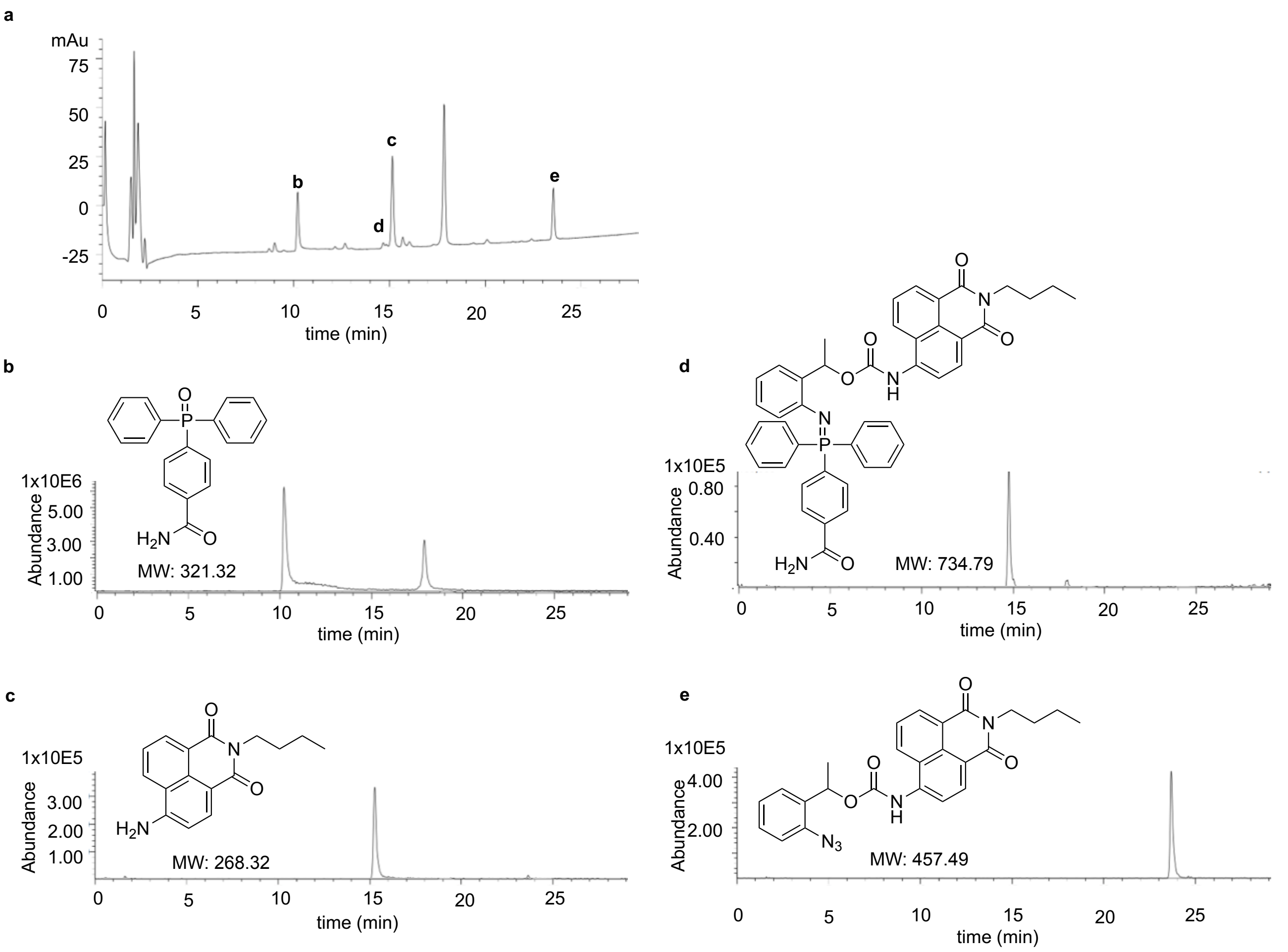

Figure S7. LC-MS analysis of reaction between azide **4** and phosphine **12.** (**a**) UV trace of reaction mixture with peaks labeled for reactants and intermediates indicated in mass traces. (**b**) Extracted LC-ESI-MS chromatogram for oxide of phosphine **12** (**c**) ABNI (**d**) aza-ylide formed by reaction of azide **4** and phosphine **12** (**e**) azide **4**.

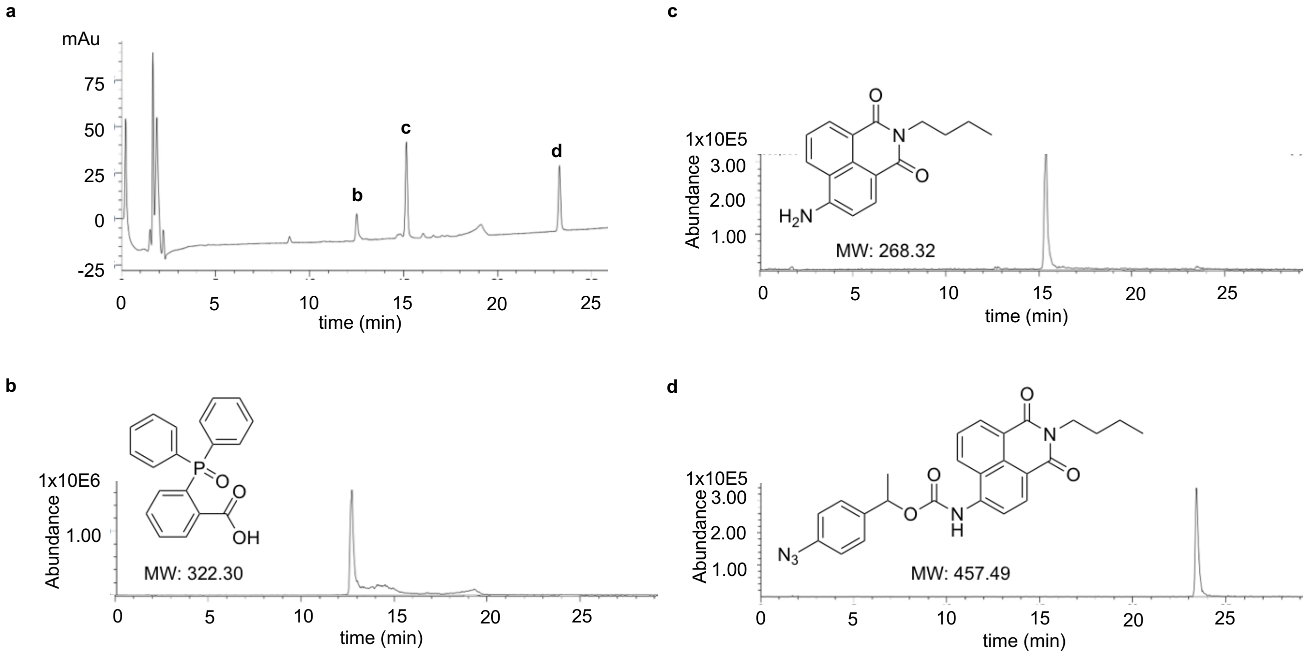

Figure S8. LC-MS analysis of reaction between azide **3** and phosphine **14.** (**a**) UV trace of reaction mixture wit peaks labeled for reactants and intermediates indicated in mass traces. (**b**) Extracted LC-ESI-MS chromatogram for oxide of phosphine **14** (**c**) ABNI (**d**) aza-ylide formed by reaction of azide **3** and phosphine **14** (**e**) azide **3**.

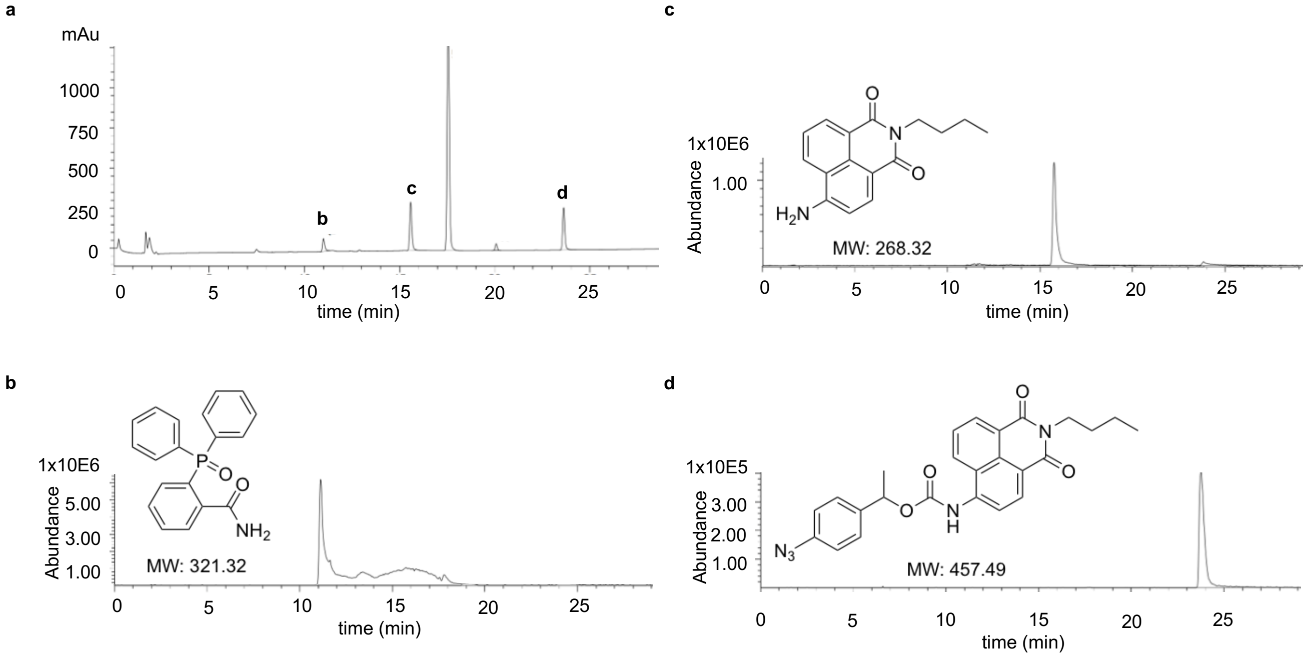

Figure S9. LC-MS analysis of reaction between azide **3** and phosphine **15.** (**a**) UV trace of reaction mixture with peaks labeled for reactants and intermediates indicated in mass traces. (**b**) Extracted LC-ESI-MS chromatogram for oxide of phosphine **15** (**c**) ABNI (**d**) aza-ylide formed by reaction of azide **3** and phosphine **15** (**e**) azide **3**.

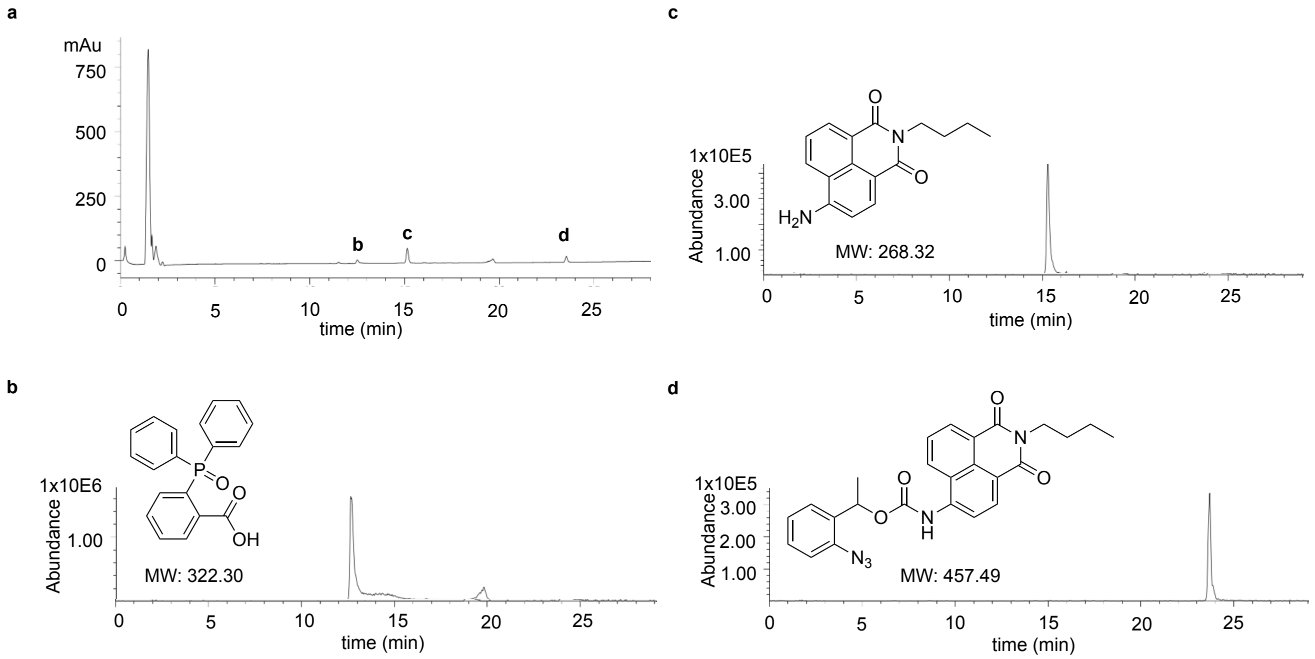

Figure S10. LC-MS analysis of reaction between azide **4** and phosphine **14.** (**a**) UV trace of reaction mixtre with peaks labeled for reactants and intermediates indicated in mass traces. (**b**) Extracted LC-ESI-MS chromatogram for oxide of phosphine **14** (**c**) ABNI (**d**) aza-ylide formed by reaction of azide **4** and phosphine **14** (**e**) azide **4**.

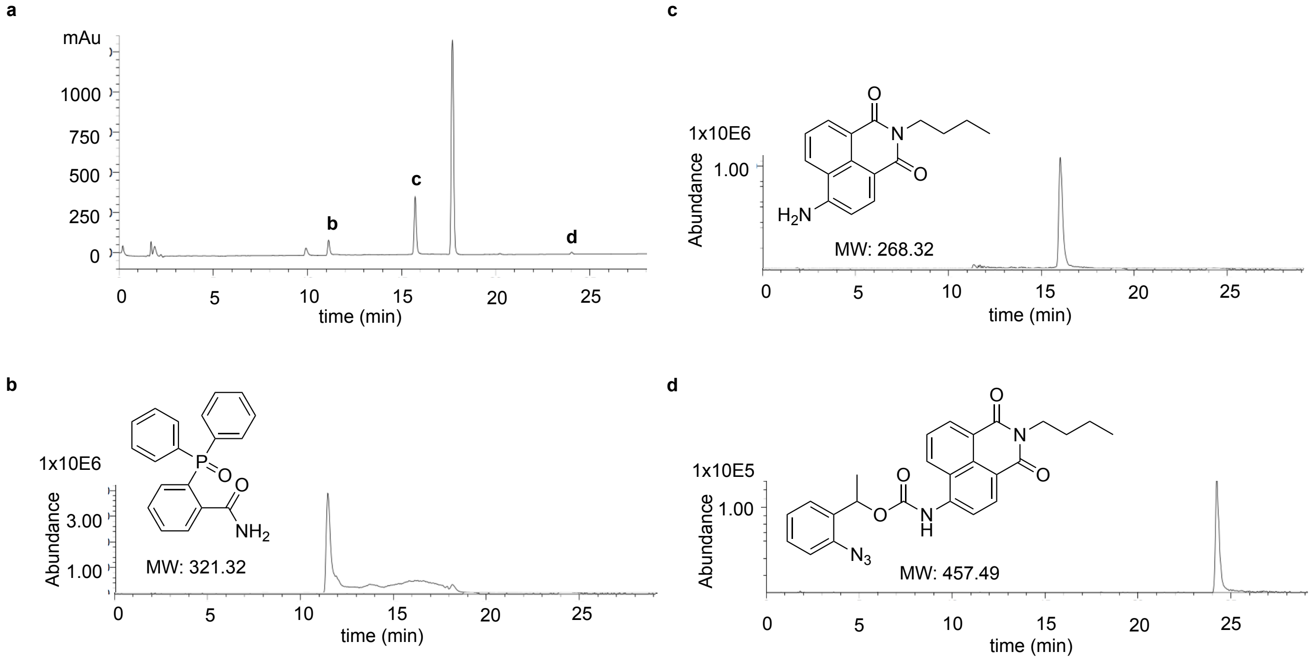

Figure S11. LC-MS analysis of reaction between azide **4** and phosphine **15.** (**a**) UV trace of reaction mixture with peaks labeled for reactants and intermediates indicated in mass traces. (**b**) Extracted LC-ESI-MS chromatogram for oxide of phosphine **15** (**c**) ABNI (**d**) aza-ylide formed by reaction of azide **4** and phosphine **15** (**e**) azide **4**.

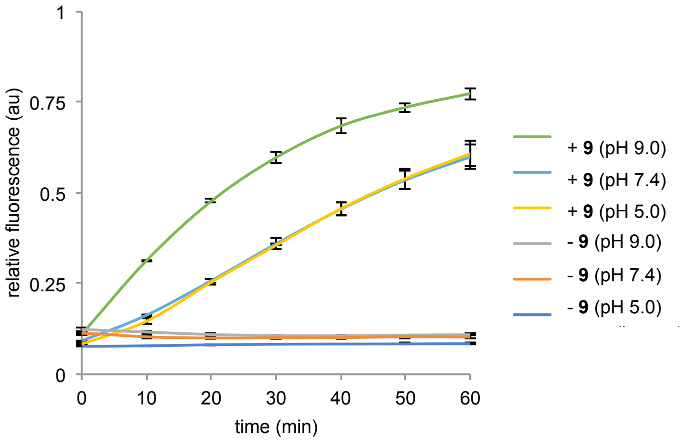

Figure S12. Study of the pH dependence of the reaction between azide **3** and phosphine **9**. Azide (10 μM) was treated with phosphine (100 μM) in 20% DMSO in citrate buffer (pH 5.0), PBS buffer (pH 7.4), or glycine-NaOH buffer (pH 9.0). Fluorescence was read every 10 minutes for 1 hour and set relative to ABNI (10 μM).

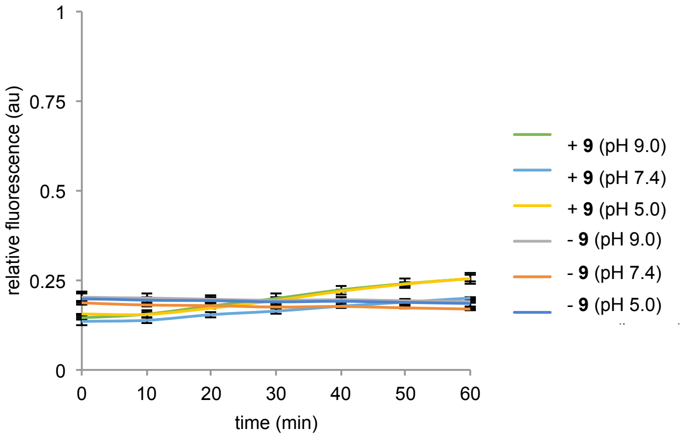

Figure S13. Study of the pH dependence of the reaction between azide **4** and phosphine **9**. Azide (10 μM) was treated with phosphine (100 μM) in 20% DMSO in citrate buffer (pH 5.0), PBS buffer (pH 7.4), or glycine-NaOH buffer (pH 9.0). Fluorescence was read every 10 minutes for 1 hour and set relative to ABNI (10 μM).

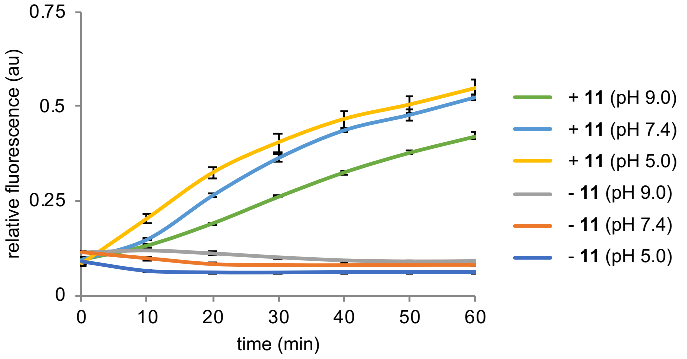

Figure S14: Study of the pH dependence of the reaction between azide **3** and phosphine **11**. Azide (10 μM) was treated with phosphine (100 μM) in 20% DMSO in citrate buffer (pH 5.0), PBS buffer (pH 7.4), or glycine-NaOH buffer (pH 9.0). Fluorescence was read every 10 minutes for 1 hour and set relative to ABNI (10 μM).

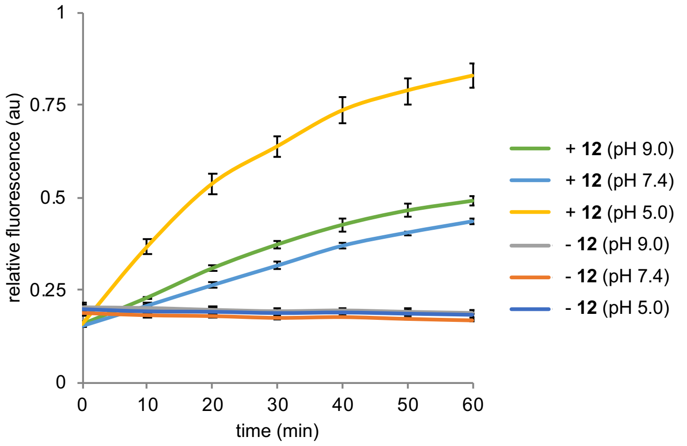

Figure S15: Study of the pH dependence of the reaction between azide **3** and phosphine **12**. Azide (10 μM) was treated with phosphine (100 μM) in 20% DMSO in citrate buffer (pH 5.0), PBS buffer (pH 7.4), or glycine-NaOH buffer (pH 9.0). Fluorescence was read every 10 minutes for 1 hour and set relative to ABNI (10 μM).

References

1. J. Wu, R. Huang, C. Wang, W. Liu, J. Wang, X. Weng, T. Tian, X. Zhou, *Org. Biomol. Chem.*, **2013**, *11*, 580-585.
2. M. Belkheira, D. El Abed, J. Pons, C. Bressy, *Chem. Eur. J.*, **2011**, *17*, 12917-12921.
3. Z. P. Demko, K. B. Sharpless, *Org. Lett.*, **2001**, *3*, 4091-4094.
4. J. T. B. Kueh, K. W. Choi, M. A. Brimble, *Org. Biomol. Chem.*, **2012**, *10*, 5993-6002.
5. K. G. Guggenheim, H. Toru, M. J. Kurth, *Org. Lett.*, **2012**, *14*, 3732-3735.
6. T. Stopka, M. Niggemann, *Chem. Commun.*, **2016**, *52*, 5761-5764.
7. H. Saneyoshi, T. Ochikubo, T. Mashimo, K. Hatano, Y. Ito, H. Abe. *Org. Lett.*, **2014**, *16*, 30-33.
8. M. Onoda, S. Uchiyama, A. Endo, H. Tokuyama, T. Santa, K. Imai, *Org. Lett.*, **2003**, *5*, 1459-1561.
